# Extracellular Matrix Remodeling and Glycoprotein Biomarkers Across Osteoarthritis, Rheumatoid Arthritis, and Temporomandibular Disorders: A Systematic Review and Integrative Analysis

**DOI:** 10.3390/ijms27198764

**Published:** 2026-09-30

**Authors:** Galina Laputková, Ivan Talian, Ján Sabo

**Affiliations:** Department of Medical and Clinical Biophysics, Faculty of Medicine, University of P. J. Šafárik, 040 11 Košice, Slovakia; galina.laputkova@upjs.sk (G.L.); jan.sabo@upjs.sk (J.S.)

**Keywords:** osteoarthritis, rheumatoid arthritis, temporomandibular disorders, glycoproteomics, glycosylation, extracellular matrix, matrix metalloproteinases, synovial fluid, saliva, biomarkers, network analysis, pathway enrichment

## Abstract

Osteoarthritis (OA), rheumatoid arthritis (RA), and temporomandibular disorders (TMDs) are heterogeneous joint diseases whose pathophysiology involves inflammation, tissue degeneration, and progressive functional decline. Although their etiologies differ, these conditions display overlapping molecular mechanisms related to extracellular matrix (ECM) remodeling, proteolytic activity, and immune signaling. This systematic review synthesizes current evidence on glycoprotein-associated and proteomic biomarkers identified in synovial fluid and saliva, predominantly derived from protein-level studies, while also evaluating the limited data on glycosylation-specific changes. Using PRISMA guidelines, thirty-eight studies met the review-level eligibility criteria. A structured molecular dataset derived from 20 of these studies was examined through integrative bioinformatics workflows, including network construction, topology analysis, Gene Ontology enrichment, UpSet intersection analysis, and pathway analysis. The analyses revealed a literature-derived disease–protein network characterized by recurrently reported extracellular matrix proteins and matrix metalloproteinases, together with enrichment of collagen-remodeling and regulated proteolytic pathways. Pathway enrichment further indicated shared mechanisms involving ECM regulation, growth-factor signaling, platelet activation, and inflammatory responses based on pathway enrichment. Glycoproteins, particularly proteoglycan 4 (PRG4), together with fibronectin (FN1), cartilage oligomeric matrix protein (COMP), and aggrecan (ACAN), were recurrently represented within the shared OA–RA protein set and were associated with extracellular matrix organization and inflammatory processes. This systematic review synthesizes protein-level biomarker evidence involving experimentally annotated glycoproteins and separately evaluates studies that directly characterized glycan or glycosylation changes. Protein-level network, overlap, and enrichment analyses identified recurrent extracellular matrix remodeling and regulation of extracellular proteolysis, particularly across OA and RA. These analyses describe disease-associated occurrence of glycoproteins and do not by themselves demonstrate altered glycosylation. Direct glycan-level characterization was available in only 5 of the 38 included studies and was largely restricted to O-glycan alterations involving proteins such as PRG4 and MMP3, underscoring the limited availability of glycosylation-specific evidence. Overall, ECM remodeling and the regulation of extracellular proteolysis emerged as the main shared molecular themes across OA and RA. Comparisons involving TMD remained exploratory because of the small evidence base and the predominance of salivary samples, which prevents separation of disease-related from biofluid-related effects. The complementary use of synovial fluid and salivary biomarkers, although requiring validation through paired-sample studies, may facilitate the development of non-invasive diagnostic and monitoring strategies.

## 1. Introduction

Osteoarthritis (OA), rheumatoid arthritis (RA), and temporomandibular disorders (TMDs) constitute a heterogeneous group of musculoskeletal diseases distinguished by chronic pain, inflammation, and progressive tissue degeneration [1,2,3]. Although these conditions share overlapping clinical features, their underlying pathophysiological mechanisms differ considerably, ranging from autoimmune-driven synovitis in RA to markedly degenerative and mechanically induced cartilage breakdown in OA with TMD manifesting a complex interaction of biomechanical and inflammatory factors. Both OA and RA often affect the temporomandibular joint (TMJ), either as part of a systemic disease or as a clinically relevant comorbidity, thereby connecting peripheral joint pathology with craniofacial dysfunction. The reported prevalence of TMJ involvement in RA varies widely (approximately 19–80%), owing to differences in disease stage and diagnostic approaches [4,5]. TMJ pathology can significantly impair mandibular function and quality of life [6,7], and osteoarthritic degeneration of the TMJ further highlights the continuum between systemic and localized joint disease [8]. Despite advances in understanding disease mechanisms, a major unresolved challenge remains the identification of robust and accessible biomarkers that enable early diagnosis, patient stratification, and monitoring of disease progression and therapeutic response [9]. The choice of biological matrix is a key factor in biomarker discovery. The synovial fluid (SF) is in direct contact with the articular tissues, thus serving as a close and specific indicator of joint pathology. Its composition mirrors local inflammatory and degradative processes. Elevated levels of cytokines, matrix metalloproteinases, and other mediators in SF are associated with disease activity in OA, RA, and TMJ disorders [9,10]. However, its clinical use is constrained by the invasive nature of sample collection, which limits its role in routine diagnostics and long-term monitoring. Unlike synovial fluid, saliva serves as a promising non-invasive biological matrix, especially in the study of temporomandibular and systemic inflammatory conditions. Its simple collection, low cost, and suitability for repeated sampling make it an appealing matrix for translational research. Accumulating evidence suggests that salivary biomarkers can reflect both systemic immune status and local pathological processes, including those associated with joint diseases [11]. However, analyzing saliva is complicated by variations in its composition, influences from oral microbiota, and the challenge of separating systemic signals from local oral factors. Glycosylation, a highly dynamic post-translational modification, contributes to the modulation of immune regulation and ECM interactions [12,13,14]. Through these mechanisms, glycan structures contribute to biomarker signatures and may influence the regulation of key biological processes underlying tissue remodeling and inflammation [15,16,17]. Several studies suggest that glycosylation alterations may contribute to disease mechanisms in addition to serving as biomarkers [13,16,18]. Previous studies have also described protein changes and post-translational modifications, particularly those involving extracellular and secreted proteins. These modifications have been recognized as a crucial component of molecular information in inflammatory joint diseases [19,20]. Glycoproteins represent a major component of the extracellular proteome and play important roles in immune regulation, matrix organization, and cell–cell communication [21]. While glycosylation can modulate protein function and stability, current evidence suggests that glycoproteomic alterations, rather than glycan structures alone, provide a more integrative and functionally relevant perspective for biomarker discovery in complex inflammatory environments [14]. A notable example is proteoglycan 4 (PRG4, lubricin), a heavily O-glycosylated glycoprotein [22,23] that is vital for joint lubrication and cartilage protection. Changes in lubricin glycosylation have been directly associated with immune-related mechanisms in OA. Truncated O-glycans on lubricin have been reported to induce the production of pro-inflammatory cytokines, including interleukin-8, C-C motif chemokine 3, and vascular endothelial growth factor A, in synovial cells, thus driving inflammatory signaling and matrix turnover [23]. Moreover, proteoglycan 4 plays a role in controlling synovial inflammation and maintaining TMJ homeostasis, indicating both structural and immunomodulatory functions [24]. Certain glycoproteins may act as mediators, linking the structural characteristics of the matrix with inflammatory signaling. In this context, glycosylation is viewed as one of several regulatory mechanisms that affect protein activity. Yet, glycosylation does not always serve as a universal driver of disease processes. The limited availability of glycan-level information compared with protein-level data highlights the value of glycoproteomic approaches in biomarker discovery.

Glycoproteins form an important functional part of the extracellular environment, contributing to structural integrity as well as signaling processes. Therefore, combining synovial fluid and saliva analyses with glycoproteomic approaches represents a relevant strategy for detecting molecular changes linked to disease and identifying clinically informative biomarkers [25,26]. In this systematic review, we sought to characterize glycoprotein-associated and proteomic biomarkers across synovial fluid and saliva in OA, RA, and TMJ disorders, with a particular focus on ECM remodeling, inflammatory pathways, and immune-related signaling.

## 2. Materials and Methods

### 2.1. Study Design and Reporting Standards

This systematic review was conducted in accordance with the PRISMA statement to ensure transparency, reproducibility, and methodological rigor [27].

### 2.2. Literature Search Strategy

A comprehensive literature search was performed on 23 February 2026 using the electronic databases PubMed, Scopus, and Web of Science. The search was restricted to studies published between 1 January 2016 and 23 February 2026. The 2016–2026 interval was selected to focus the systematic synthesis on contemporary proteomic, glycoproteomic, and biomarker studies and their current analytical frameworks. Earlier studies were therefore outside the prespecified evidence window, although relevant foundational work was considered in the mechanistic context of the Introduction and Discussion. The search strategy combined controlled vocabulary and free-text terms related to biological fluids (synovial fluid and saliva), inflammation and biomarkers, glycosylation and proteomics, and musculoskeletal diseases (osteoarthritis, rheumatoid arthritis, and temporomandibular disorders). Boolean operators (“AND”, “OR”) were used to structure the query.

The following search string was applied in PubMed: (synovial fluid[Title/Abstract] OR saliva[Title/Abstract]) AND (inflammatory mediator*[Title/Abstract] OR inflammation[Title/Abstract] OR inflammatory[Title/Abstract] OR immunomodulation[Title/Abstract] OR biomarker*[Title/Abstract]) AND (glycan*[Title/Abstract] OR glycoprotein*[Title/Abstract] OR glycoproteomic*[Title/Abstract] OR proteomic*[Title/Abstract] OR PRG4[Title/Abstract] OR fibronectin[Title/Abstract] OR tenascin[Title/Abstract] OR galectin-8[Title/Abstract] OR osteopontin[Title/Abstract] OR TNF[Title/Abstract] OR cytokin*[Title/Abstract] OR interleukin*[Title/Abstract]) AND (osteoarthritis OR rheumatoid arthritis OR TMJ OR temporomandibular disorders).

Equivalent keyword adaptations were applied for Scopus and Web of Science to ensure consistency across databases.

### 2.3. Eligibility Criteria

#### 2.3.1. Inclusion Criteria

Studies were included if they satisfied the following conditions: (i) involved human-derived saliva and/or synovial fluid; (ii) examined inflammatory mechanisms, immunomodulatory activity, or biomarkers; (iii) incorporated glycan, glycoprotein, proteomic, or glycoproteomic analyses; (iv) reported at least one relevant molecule, such as proteoglycan-4, fibronectin, tenascin, galectin-8, osteopontin, tumor necrosis factor (TNF), cytokines, or interleukins; (v) focused on OA, RA, TMD, or TMJ pathology, and (vi) were published in English between 2016 and 2026.

Studies examining OA, RA, or TMD in the context of a comorbid condition were eligible when the target joint-disease population, biological matrix, and biomarker measurements otherwise satisfied the inclusion criteria; the comorbid condition was treated as a stratifying or comparator variable rather than as an additional disease category.

#### 2.3.2. Exclusion Criteria

Studies were excluded according to the following criteria: (i) non-human research (animal or in vitro-only); (ii) investigations focusing on isolated cells without considering the context of whole saliva or synovial fluid; (iii) review papers, conference abstracts, editorials, letters, or case reports; (iv) retracted articles; and (v) studies that did not address glycosylation, glycoproteins, proteomics, or inflammatory biomarkers within the specified disease scope. Before manual screening, database filtering tools were applied to remove duplicate records, non-English publications, non-human studies, and publication types that failed to meet the predetermined eligibility criteria.

The presence of a healthy or disease comparator group was not required for study-level eligibility; comparator adequacy was instead considered as part of the risk-of-bias assessment.

### 2.4. Study Selection Process

All records obtained from the databases were exported and combined in a reference management system, and duplicate entries were deleted. The screening process was carried out independently by two reviewers. At first, titles and abstracts were checked according to the predefined eligibility criteria. Full-text articles were then retrieved for studies that satisfied the inclusion criteria or when eligibility was still uncertain. Any disagreements between reviewers were settled through discussion and consensus. In addition, the reference lists of all included studies were manually reviewed to find other relevant publications that were not captured in the initial database search.

### 2.5. Data Collection and Synthesis

All 38 studies contributed to the qualitative synthesis and risk-of-bias assessment. For the downstream integrative analyses, molecular data were extracted from the 20-study analytical subset using a structured framework covering study characteristics, biological matrix, target molecules, analytical methods, and disease context.

The collected data were then systematically organized and qualitatively summarized to identify protein-level biomarker patterns and, where available, glycosylation-specific findings. For the downstream integrative analyses, proteins were restricted to molecules with experimentally supported glycosylation annotations in UniProt; this criterion identifies proteins as glycoproteins but does not imply disease-associated alteration of their glycosylation state.

### 2.6. Study Selection Flow Diagram

**Figure 1:**
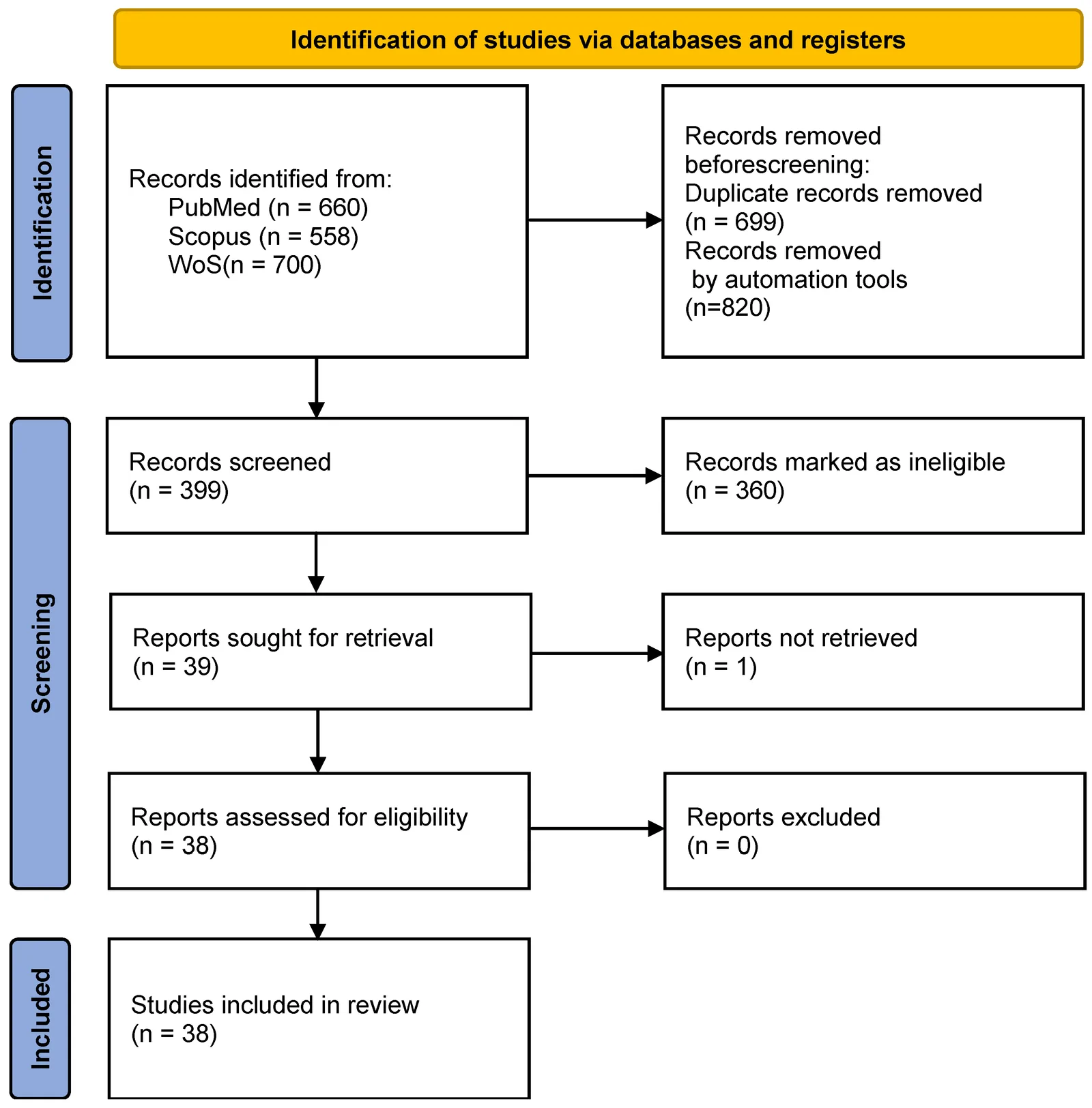
The flow diagram of study selection. PRISMA 2020 flow chart template sourced from Page et al. [28].

The study selection process is summarized in a PRISMA flow diagram (Figure 1), detailing the number of records identified, screened, excluded, and included at each stage of the review. Studies were considered eligible for the systematic review according to the criteria specified in Section 2.3. Eligibility for the systematic review was assessed independently of subsequent inclusion of individual molecular observations in the integrative analyses.

### 2.7. Risk of Bias Assessment

The methodological quality of the included studies was assessed using a modified version of the ROBINS-I (Risk Of Bias In Non-randomized Studies of Interventions) tool, adjusted for observational biomarker and omics-based research [29]. The following seven domains were considered: (D1) bias due to confounding, (D2) bias in participant selection, (D3) bias in classification of exposure or disease status, (D4) bias from deviations in intended exposure or sampling processes, (D5) bias due to missing data, (D6) bias in outcome measurement, and (D7) bias in selection of the reported result. Each domain was rated as having low, moderate, or serious risk of bias, and an overall risk-of-bias judgment was assigned to each study based on the highest level of bias identified across domains. The evaluation relied on a detailed review of study design, population characteristics, biomarker measurement methods, statistical analysis, and the clarity of reporting. Because the included studies were mainly cross-sectional, case–control, and exploratory omics analyses, special consideration was given to confounding factors (e.g., age, sex, disease severity, comorbidities), participant selection methods, and the presence of prespecified analytical strategies. The outcomes of the risk-of-bias assessment were displayed using a traffic-light plot, following the ROBINS-I guidelines.

### 2.8. Risk-of-Bias Sensitivity Analysis

To determine whether the integrative findings were disproportionately influenced by studies with serious methodological limitations, a sensitivity analysis was performed after excluding studies assigned an overall serious risk-of-bias judgment. Disease-associated protein sets were reconstructed from the remaining studies using the same identifier harmonization and UniProt glycosylation-annotation criteria as in the primary analysis. The weighted disease–protein network, topological measures, GO overrepresentation analysis, and Reactome pathway analysis were then recalculated using identical analytical parameters. Robustness was evaluated by comparing protein-set overlap, weighted-node rankings, and the retention or loss of significantly enriched functional categories and pathways between the primary and sensitivity analyses.

## 3. Results

### 3.1. Identification of Studies and Characteristics of Included Studies

The study selection process is summarized in Figure 1. A total of 1918 records were initially identified through database searching. After removing duplicates and automated exclusions, 399 records were screened by titles and abstracts. Among these, 360 records were excluded during this stage because they did not meet the predefined eligibility criteria. The full texts of 39 reports were sought for retrieval, of which 38 were successfully obtained and assessed for eligibility, while one report could not be retrieved. Following full-text reassessment against the predefined eligibility criteria, no reports were excluded; all 38 assessed reports satisfied the review-level eligibility criteria and were included in the systematic review. The characteristics of the 38 included studies are presented in Supplementary Table S1. The selected works covered investigations of biomarker profiles in OA, RA, and TMD using synovial fluid, saliva, or both as biological matrices. Most research in OA has focused on synovial fluid. This focus is largely due to the anatomical proximity of synovial fluid to the affected joint and its key role in assessing localized pathological changes. In contrast, saliva was more often examined in RA and TMD, highlighting its potential as a non-invasive source of biomarkers linked to systemic and joint-related inflammation. Methodologically, most studies used targeted immunoassay-based approaches, including enzyme-linked immunosorbent assays (ELISAs) and multiplex platforms, to measure specific proteins. A smaller group applied mass spectrometry-based proteomics and glycoproteomics, which allowed a broader characterization of extracellular proteins and their post-translational modifications. Only a few studies offered detailed glycan-level data, mainly examining O-glycosylation patterns of selected proteins such as proteoglycan 4 and matrix metalloproteinase-3. Among the included studies, the most commonly reported molecules were matrix metalloproteinases (MMP1, MMP3, MMP8, and MMP9), proteins found in ECM (e.g., cartilage oligomeric matrix protein (COMP), lumican), and inflammatory mediators (e.g., interleukin-8, galectin-3). ECM turnover and proteolytic activity appeared to be the most frequently examined biological processes in these studies. In addition, several proteins, particularly lubricin (PRG4), was among the recurrently reported ECM-associated proteins. Overall, ECM remodeling, inflammatory signaling, and proteolytic activity were the processes most often linked with disease across studies.

### 3.2. Risk of Bias Assessment

**Figure 2:**
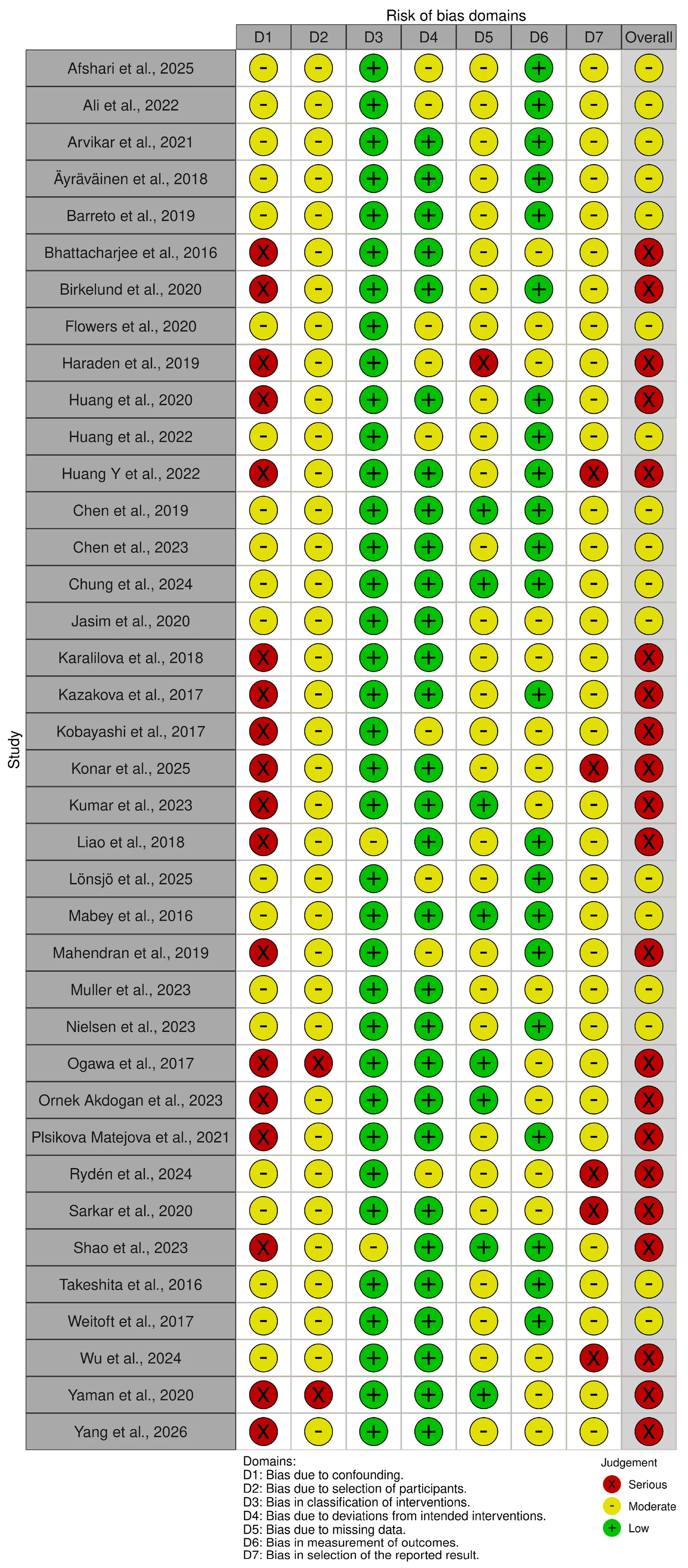
Risk-of-bias assessment of included studies using the modified ROBINS-I method, adapted for observational biomarker and omics studies [29]. Traffic-light plot summarizing the domain-level and overall risk-of-bias judgments for each included study. The assessed domains included bias due to confounding (D1), selection of participants (D2), classification of exposure or disease status (D3), deviations from intended exposure or sampling procedures (D4), missing data (D5), measurement of outcomes (D6), and selection of the reported result (D7). Each domain was rated as low (green), moderate (yellow), or serious (red) risk of bias. Overall risk-of-bias judgments were determined according to the modified ROBINS-I framework, whereby the overall rating corresponded to the highest risk level observed across the assessed domains. Most studies demonstrated moderate to serious risk of bias, primarily driven by confounding, participant selection, and selective reporting [23,30,31,32,33,34,35,36,37,38,39,40,41,42,43,44,45,46,47,48,49,50,51,52,53,54,55,56,57,58,59,60,61,62,63,64,65,66].

The risk-of-bias assessment (Figure 2, Supplementary Table S2) indicated that all included studies had either moderate or serious risk of bias across the assessed domains. Overall, 17 studies were judged to have moderate risk of bias and 21 studies were judged to have serious risk of bias.

The most common sources of bias were confounding (D1), mainly due to insufficient adjustment for key clinical and biological variables such as age, disease severity, metabolic status, and comorbid conditions, and selection of the reported result (D7), reflecting the exploratory nature of many biomarker studies and the lack of prespecified analytical strategies, which increases the likelihood of selective reporting. Selection bias (D2) was also often noted in studies that relied on convenience sampling, small pilot cohorts, or non-equivalent control groups, limiting comparability between study populations. Bias related to classification of disease or exposure (D3) was generally low since most studies used established diagnostic criteria. Similarly, bias due to deviations from intended sampling or experimental procedures (D4) was limited, linked to the use of standardized laboratory workflows. Still, moderate concerns persisted in some studies because of heterogeneous sample sources or inconsistencies in control definitions.

Missing data (D5) were typically moderate, particularly in studies with incomplete clinical metadata or reduced sample availability after laboratory processing. Measurement bias (D6) was also generally moderate and mainly reflected variability in analytical platforms, limited technical validation, and the absence of assessor blinding. Overall, all included studies were judged to have either moderate or serious risk of bias, with serious risk being more frequent (21/38 studies) than moderate risk (17/38 studies), primarily because of concerns related to confounding, participant selection, and selective reporting.

### 3.3. Disease–Protein Network Analysis

**Figure 3:**
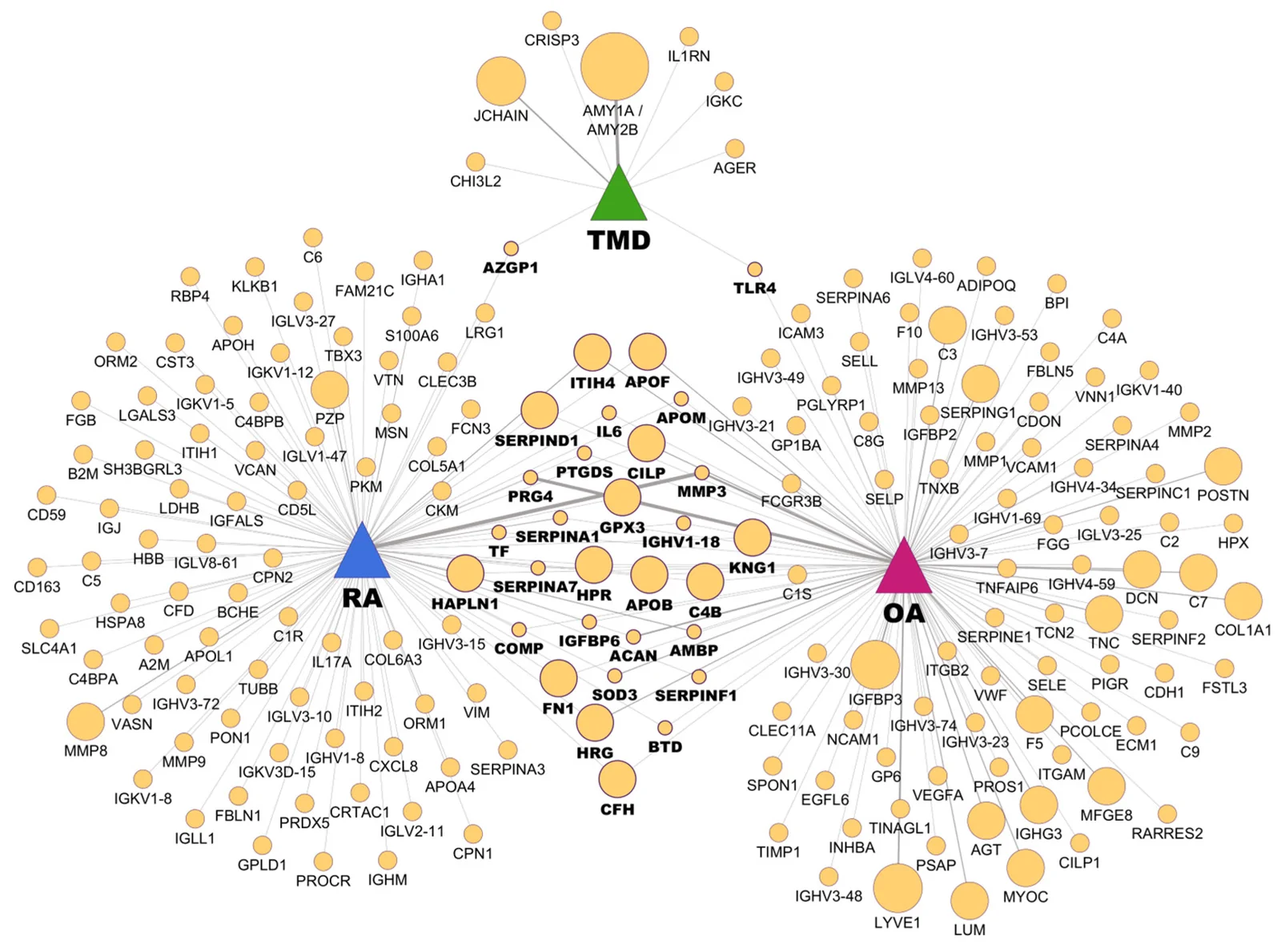
Disease–protein network of osteoarthritis (OA), rheumatoid arthritis (RA), and temporomandibular disorders (TMDs) constructed in Cytoscape (v3.10.4). Triangular nodes indicate diseases, while elliptical nodes represent proteins identified in synovial fluid or saliva studies. Disease nodes are colored by diagnosis, and protein nodes appear in orange. The size of each node reflects how often a protein was reported across the 20-study analytical subset, with larger nodes marking proteins found more frequently. Edge width shows the number of times a protein–disease association was observed in these studies. The network highlights both disease-specific and shared protein patterns, revealing substantial overlap between OA and RA, especially for extracellular matrix, complement, inflammatory, and cartilage-remodeling proteins, whereas the smaller TMD dataset is composed predominantly of salivary proteins; direct interpretation of this difference as disease-specific is limited by the unequal distribution of biofluid types.

A total of 115 unique proteins/gene symbols for OA, 106 for RA, and 9 for TMD were extracted from the 20-study analytical subset after removal of duplicate entries. Of the 115 proteins related to OA, 85 were unique to OA, while the remaining proteins were also identified in other disease groups. Distinct yet partly overlapping protein patterns linked to OA, RA, and TMD are shown on the disease–protein network (see Figure 3). OA was associated with the largest number of proteins in the bipartite network, including numerous ECM, inflammatory, complement, and tissue-remodeling proteins such as COMP, DCN, LUM, LYVE1, MFGE8, POSTN, COL1A1, C3, C4B, C7, C8G, C9, MMP1, MMP2, MMP3, TIMP1, and TNFAIP6. Several proteins displayed large node sizes, reflecting frequent reporting across studies, including IGFBP3, LYVE1, COMP, POSTN, and DCN. The RA-centered subnetwork consisted of proteins involved in immune regulation, complement activation, acute-phase responses, and cartilage degradation, including PRG4, GPX3, HRG, SOD3, FN1, ACAN, CILP, COMP, MMP3, MMP8, MMP9, SERPINA1, SERPINA7, and several complement components. Among these, PRG4, COMP, GPX3, HRG, ACAN, and MMP3 represented major nodes within the network linked to RA. The TMD cluster, in contrast, was smaller and more focused, dominated by salivary proteins such as AMY1A/AMY2B and JCHAIN, which reflects the limited set of proteins represented in the 20-study analytical subset. In the combined datasets for OA and RA, twenty-nine proteins were identified that formed the common core of the network, including COMP, ACAN, FN1, CILP, GPX3, HRG, SERPINA1, MMP3, and PRG4. These proteins participated in ECM turnover, cartilage integrity, complement activation, lubrication, and inflammatory processes. They included multiple complement components, matrix metalloproteinases, serpins, and ECM proteins. In contrast, a single protein, TLR4, was shared between OA and TMD, while AZGP1 appeared in both RA and TMD. OA and RA showed a considerable degree of overlap in their protein patterns, whereas the TMD dataset was considerably smaller and was dominated by proteins identified in saliva. The TMD dataset therefore appeared compositionally different from the larger OA and RA datasets. However, this difference cannot be attributed confidently to TMD-specific biology because the TMD evidence was derived predominantly from saliva, whereas OA and much of the RA evidence were derived from synovial fluid. Disease-related and biofluid-related effects are therefore confounded in these comparisons.

The literature-derived network encodes the presence and recurrence of reported disease–protein associations but not the direction or magnitude of protein abundance changes. Consequently, network edges do not distinguish increased from decreased protein abundance and should not be interpreted as directional regulatory relationships.

### 3.4. UpSet Analysis

**Figure 4:**
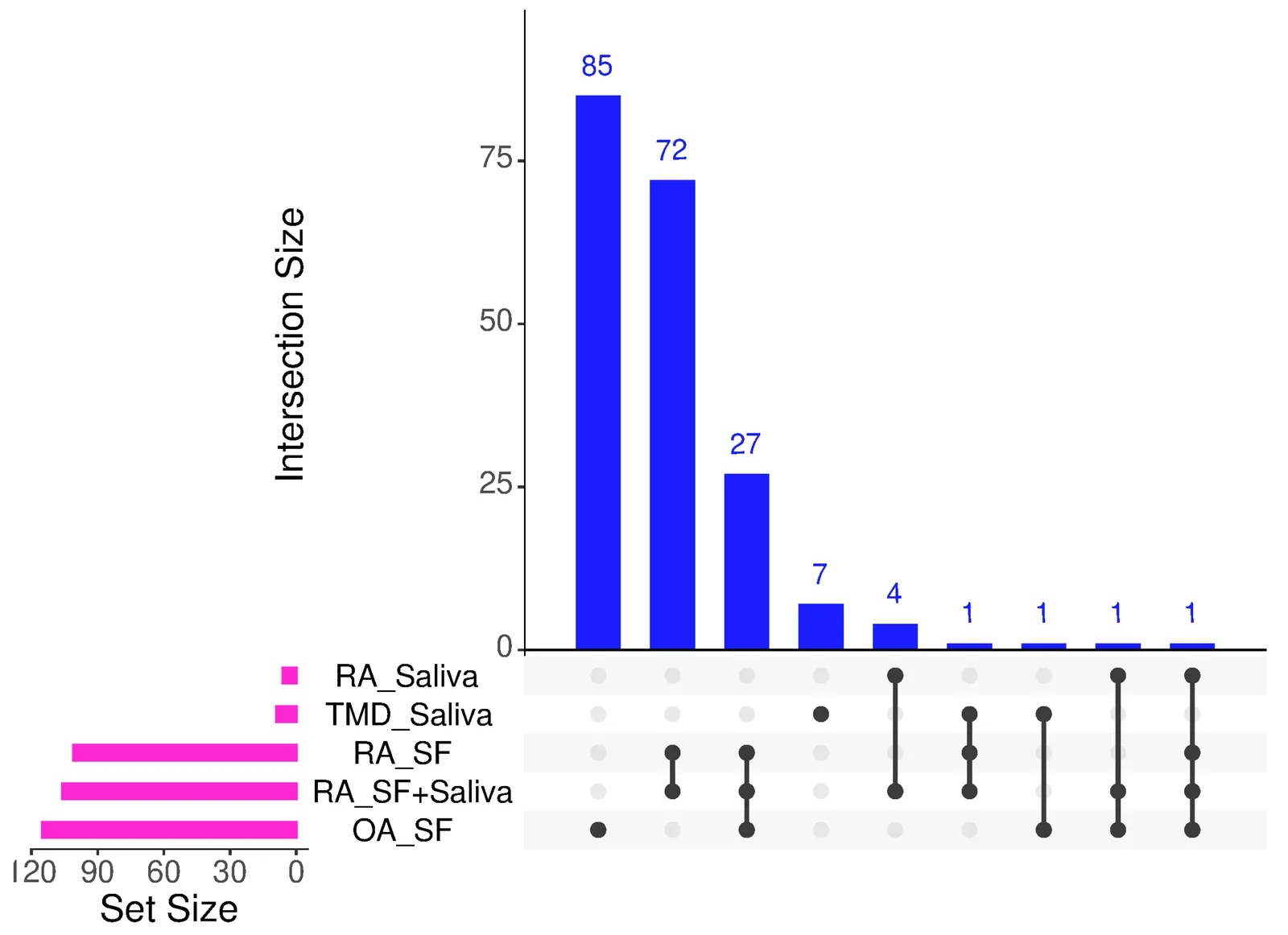
UpSet plot showing the overlap of proteins among osteoarthritis (OA) synovial fluid, rheumatoid arthritis (RA) synovial fluid, RA saliva, and temporomandibular disorder (TMD) saliva datasets. Horizontal bars on the left indicate the total number of proteins identified in each dataset. Vertical bars represent the size of each intersection, while connected dots below indicate the datasets contributing to a given intersection. The largest unique protein sets were observed for OA synovial fluid (*n* = 85) and RA synovial fluid and saliva (*n* = 72). Twenty-seven proteins were shared between OA synovial fluid and RA (synovial fluid and saliva) datasets, representing the largest overlap. Smaller intersections included proteins common to RA saliva and RA datasets (*n* = 4); OA synovial fluid and TMD saliva (*n* = 1); OA synovial fluid, RA saliva, RA synovial fluid and saliva (*n* = 1); RA synovial fluid, RA synovial fluid and saliva, TMD saliva (*n* = 1); and OA synovial fluid, RA synovial fluid, RA saliva, RA synovial fluid and saliva (*n* = 1).

UpSet analysis was conducted to assess protein overlaps among OA synovial fluid, RA synovial fluid, RA saliva, RA synovial fluid and saliva, and TMD saliva datasets (Figure 4). Unlike the disease–protein network that used combined OA and RA datasets, the Upset analysis treated biofluid-specific datasets separately. OA synovial fluid showed the largest disease-specific protein set (85 proteins), followed by RA synovial fluid (72 proteins), while TMD saliva and RA saliva contained only seven and four unique proteins, respectively. The greatest overlap appeared between OA and RA synovial-fluid datasets, consisting of 29 shared proteins, such as ACAN, COMP, PRG4, MMP1, MMP2, MMP3, SERPINA1, TIMP1, and VCAN, which are linked to ECM organization, cartilage integrity, and inflammatory processes. In contrast, the overlap among salivary datasets was limited, with only four proteins shared between RA saliva and RA synovial fluid, and single shared proteins identified between OA and TMD (TLR4) and between OA and RA saliva (IL6). SERPINA1 was the only biomarker detected in OA, RA synovial fluid, and RA saliva at the same time. The absence of proteins shared across all OA, RA synovial fluid, RA saliva, and TMD saliva datasets highlights considerable heterogeneity among the disease–biofluid strata; however, the present dataset does not allow disease-related effects to be separated reliably from biofluid-related effects. The difference compared with the disease network reflects that the network analysis used combined OA and RA datasets, while the UpSet analysis examined biofluid-specific datasets separately.

### 3.5. Disease–Protein Weighted Network Analysis

**Figure 5:**
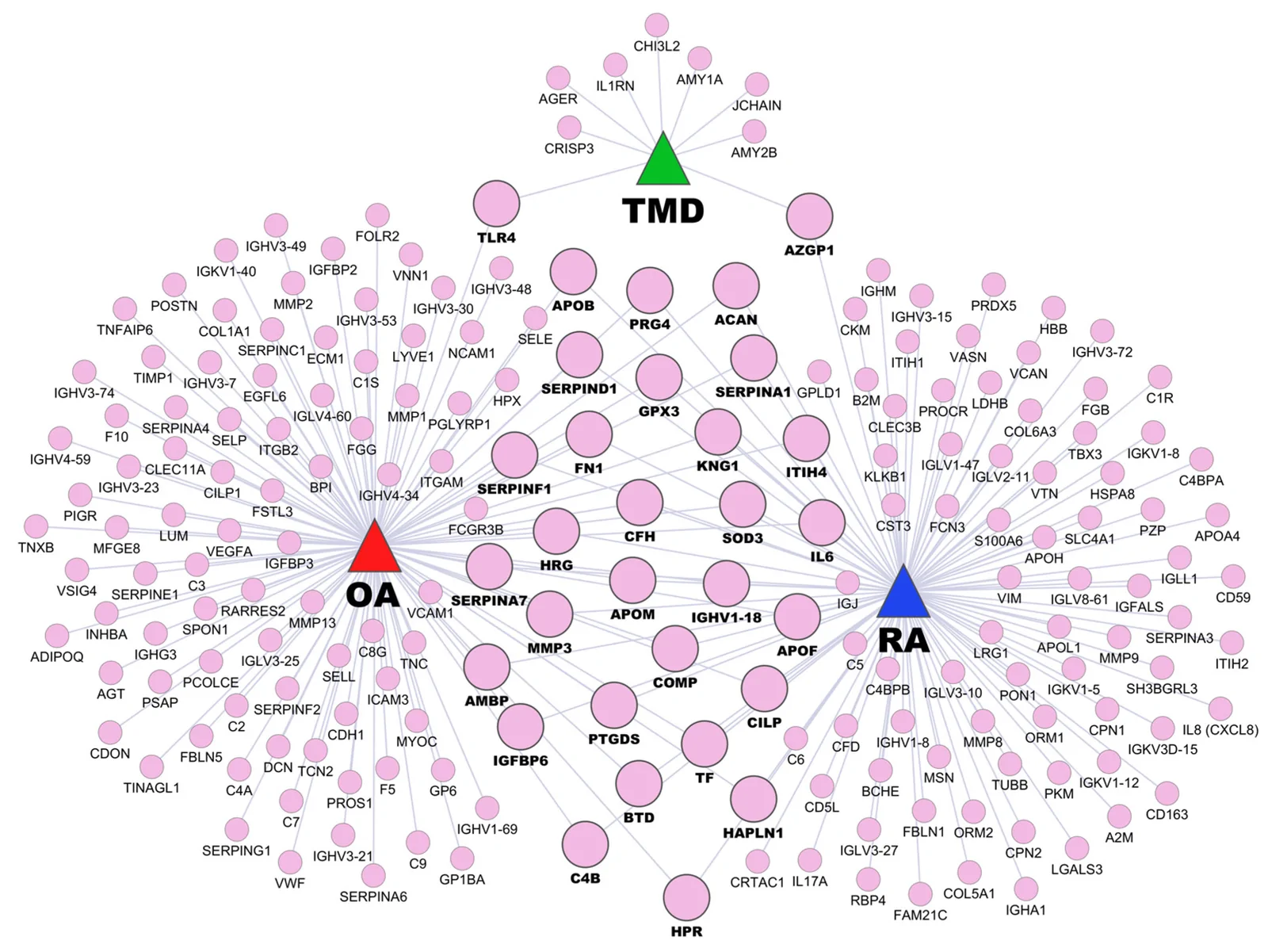
Weighted disease–protein bipartite network of proteins identified in osteoarthritis (OA), rheumatoid arthritis (RA), and temporomandibular disorders (TMDs). Disease nodes appear as colored triangles, while proteins are shown as circles. Node size reflects the weighted degree (node strength), calculated as the total of edge weights connected to each protein. Edge width represents the number of studies reporting each disease–protein association. The network shows a large, shared OA–RA cluster enriched in ECM proteins, protease inhibitors, complement components, and inflammatory mediators, while the considerably smaller TMD cluster is composed predominantly of salivary proteins, limiting direct comparison with the largely synovial-fluid-derived OA and RA datasets. Proteins with high weighted degree or cross-disease connectivity, including MMP3, PRG4, ACAN, COMP, IL6, SERPINA1, TLR4, and AZGP1, represent recurrently reported or cross-disease proteins within the literature-derived network.

The weighted disease–protein bipartite network (Figure 5) analyzed using the Network Analyzer in Cytoscape consisted of 202 nodes and 230 edges, linking three disease nodes (OA, RA, and TMD) with disease-associated proteins identified from the literature. The network showed a low density (0.011) and a clustering coefficient of zero, which is expected for a bipartite disease–protein structure where proteins connect mainly to diseases rather than to each other. The relatively high network heterogeneity (4.765) and network centralization (0.566) indicate an uneven distribution of associations within the literature-derived network, with a subset of proteins showing greater recurrence or cross-disease connectivity. The network contained a notable OA–RA protein cluster, indicating considerable overlap between the two arthritic conditions. In the network representation, the TMD cluster formed a smaller and more isolated subnetwork connected through only a few proteins, reflecting the smaller and predominantly saliva-derived TMD dataset. The average path length (2.79) and network diameter (4) suggest that most proteins are separated by only a few network steps through the disease nodes, reflecting the compact nature of the network. Several proteins showed high degree centrality due to their associations with multiple disease groups. Proteins with the highest degree centrality included ACAN, AMBP, APOB, APOF, APOM, COMP, FN1, IL6, MMP3, PRG4, SERPINA1, TLR4, and AZGP1, along with several other ECM and plasma-derived proteins. Many of these proteins belonged to the shared OA–RA set, whereas TLR4 and AZGP1 connected OA with TMD and RA with TMD, respectively. Proteins with high degree or weighted degree were associated with extracellular matrix/cartilage (ACAN, COMP, PRG4, CILP, HAPLN1, FN1), matrix degradation/remodeling (MMP3), inflammation/immune signaling (IL6, TLR4, CFH), lipid transport and metabolism (APOB, APOF, APOM), protease inhibition/complement (SERPINA1, SERPINA7, SERPIND1, SERPINF1), and plasma-derived proteins (HRG, HPR, TF, KNG1). ACAN, COMP, PRG4, FN1, and CILP occupied central positions in the OA–RA shared protein cluster. IL6 and TLR4 were among the most connected inflammatory proteins. Betweenness centrality analysis identified TLR4 (0.040) and AZGP1 (0.037) as the proteins with the highest betweenness centrality within the evidence network. Proteins shared between OA and RA, including ACAN, COMP, FN1, IL6, MMP3, PRG4, and SERPINA1, displayed intermediate betweenness values (0.012) and high neighborhood connectivity (110.5), consistent with their position within the large shared OA–RA biomarker cluster. OA-specific and RA-specific proteins had neighborhood connectivity values of 115 and 106, respectively, while proteins identified in TMD showed much lower values (9), due to the smaller size and relative isolation of the TMD subnetwork. Analysis of edge weights, representing the number of independent reports supporting each disease–protein association, identified MMP3 as the most frequently reported biomarker (weight = 7). Among proteins identified in TMD, AMY1A and AMY2B had high weights (6 each), consistent with their repeated identification in salivary studies. Other highly supported biomarkers included SERPINA1, IGFBP3, LYVE1, and JCHAIN (weight = 3), while proteins such as AMBP, ITIH4, AGT, C3, C7, COL1A1, DCN, F5, LUM, MFGE8, POSTN, SERPING1, TNC, MMP8, and PZP were supported by two independent studies. SERPINA1 was one of the proteins found across several disease datasets and biological fluids. TLR4 and AZGP1 linked the TMD cluster with the OA and RA subnetworks, respectively, indicating a partial overlap between salivary TMD biomarkers and proteins associated with arthritic conditions.

### 3.6. Gene Ontology Enrichment Analysis

**Figure 6:**
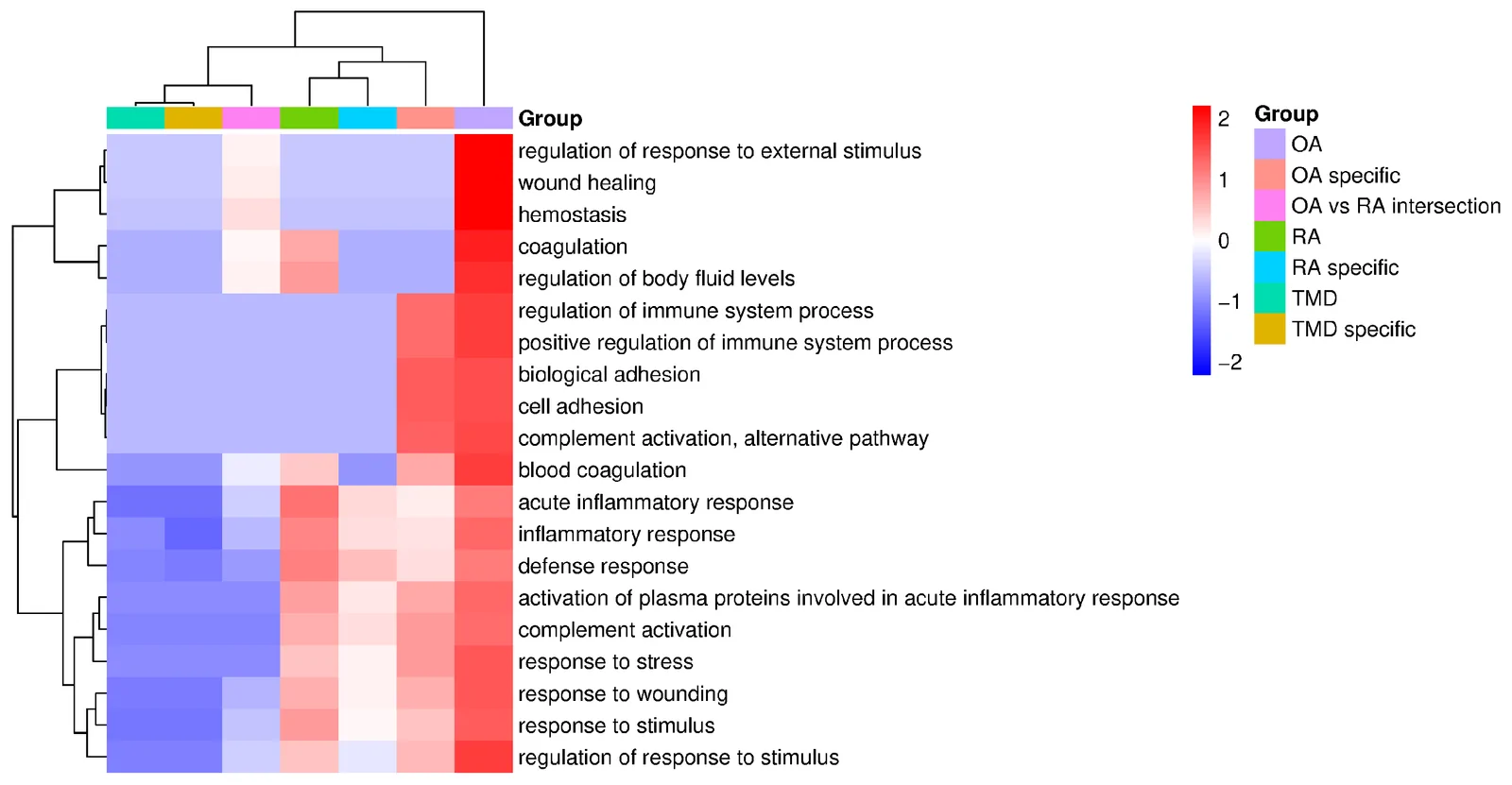
Gene Ontology Biological Process (GO-BP) enrichment heatmap. Hierarchical clustering heatmap displaying the top 20 enriched Gene Ontology Biological Process (GO-BP) terms across osteoarthritis (OA), rheumatoid arthritis (RA), temporomandibular disorders (TMDs), disease-specific protein sets (OA-specific, RA-specific, TMD-specific), and proteins shared between OA and RA (OA–RA intersection). Values represent row-scaled −log10(FDR) enrichment scores (Z-score transformed). Clustering revealed a group of inflammatory-response terms, including acute inflammatory response, inflammatory response, defense response, response to wounding, and complement activation, which are most strongly enriched in RA, RA-specific proteins, and the OA–RA intersection. Another cluster comprising cell adhesion, biological adhesion, and regulation of immune system processes is mainly associated with OA-specific proteins and reflects extracellular matrix-related and adhesion-related biological processes. Dendrograms show functional similarities among disease groups and enriched biological processes.

**Figure 7:**
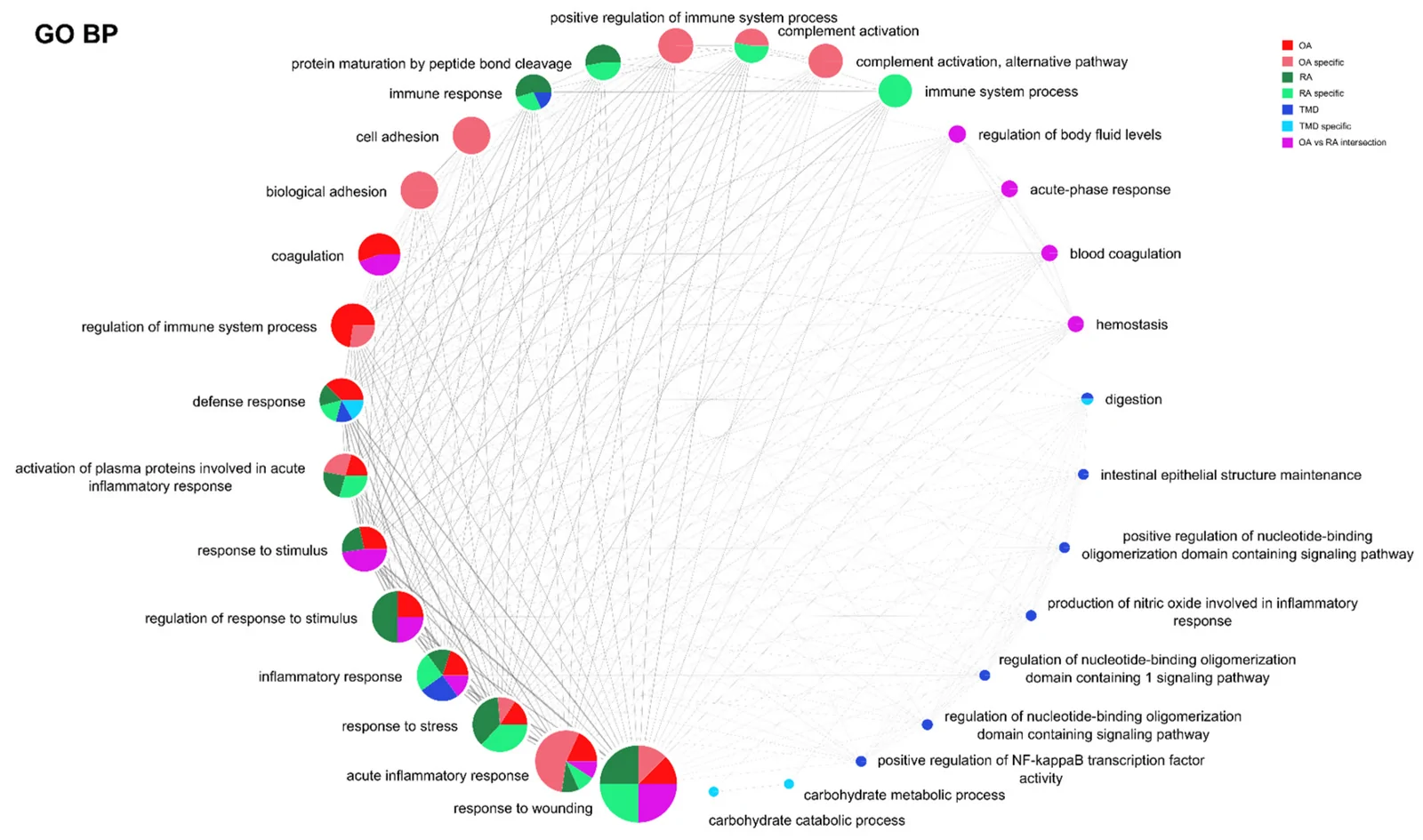
Gene Ontology Biological Process (GO-BP) enrichment map network. Enrichment map network showing the top ten enriched Gene Ontology Biological Process (GO-BP) terms identified in osteoarthritis (OA), rheumatoid arthritis (RA), temporomandibular disorders (TMDs), disease-specific protein sets (OA-specific, RA-specific, TMD-specific), and proteins shared between OA and RA (OA–RA intersection). Each node represents an enriched GO term, and its size is proportional to enrichment significance (−log10(FDR)). Pie-chart sectors display the relative contribution of each disease group to the enrichment of a given term. Edges link GO terms that share common genes, with edge width corresponding to the number of shared genes. The network shows a highly interconnected group of inflammatory-response terms, including response to stimulus, response to wounding, defense response, and acute inflammatory response, shared by OA and RA. OA-specific terms form a distinct cluster related to cell adhesion, biological adhesion, and complement activation, while terms associated with proteins unique to the TMD dataset cluster around digestion, carbohydrate metabolism, and innate immune signaling; given the predominance of salivary samples in the TMD dataset, these patterns may reflect biofluid composition as well as disease-related biology. The strong connectivity among inflammatory and coagulation-related processes indicates substantial gene overlap among these enriched terms.

**Figure 8:**
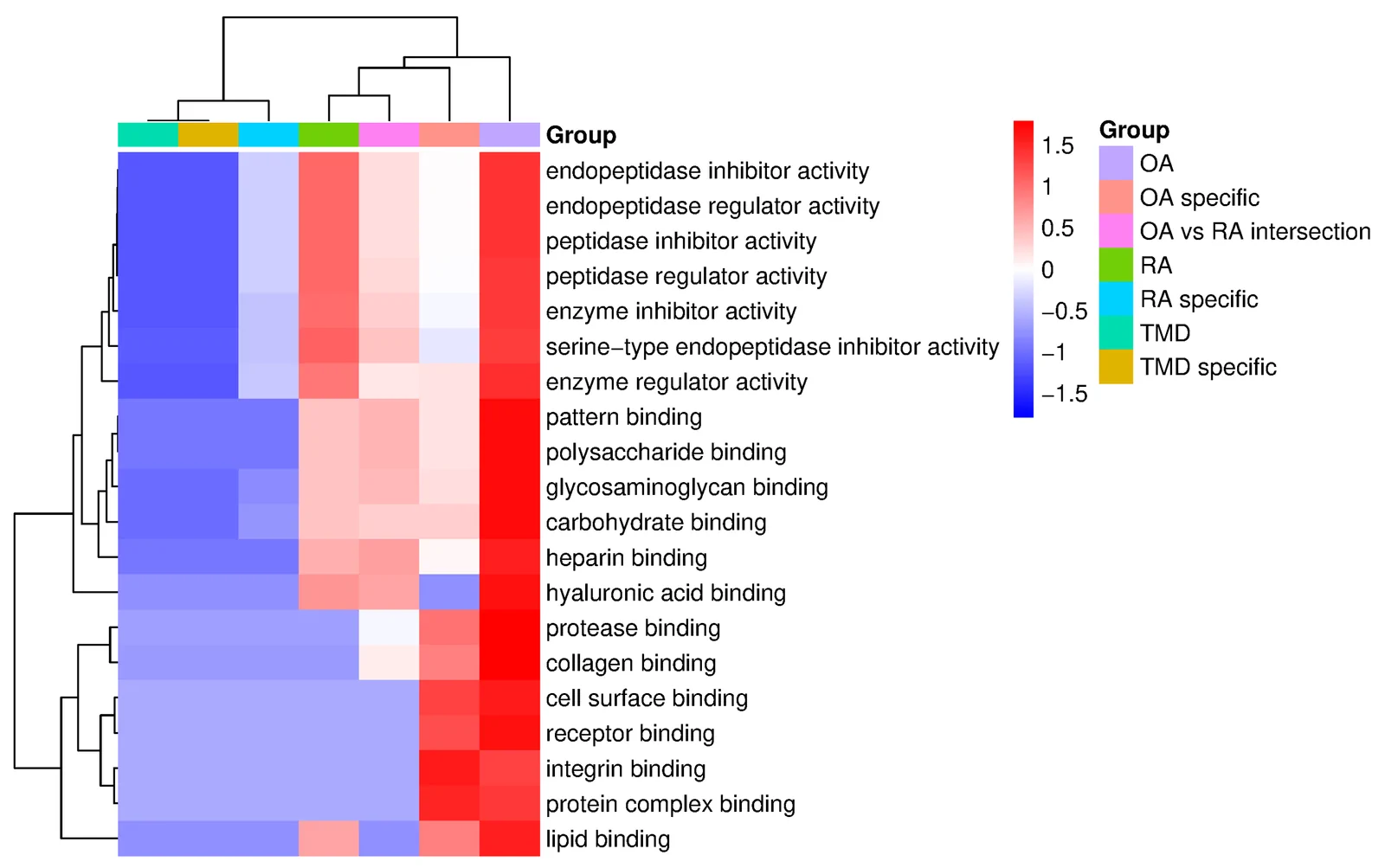
Gene Ontology Molecular Function (GO-MF) enrichment heatmap. Hierarchical clustering heatmap of the top 20 enriched Gene Ontology Molecular Function (GO-MF) terms identified in OA, RA, TMD, disease-specific protein sets, and the OA–RA intersection. Values show row-scaled −log10(FDR) enrichment scores (Z-score transformed). The heatmap highlights a marked enrichment of endopeptidase inhibitor activity, peptidase regulator activity, and serine-type endopeptidase inhibitor activity within RA datasets and the OA–RA intersection. This pattern aligns with the regulation of extracellular proteolysis during inflammation. OA-specific proteins display enrichment for extracellular matrix-related binding functions, such as collagen binding, integrin binding, receptor binding, and protein complex binding. Shared OA–RA proteins show notable enrichment of glycosaminoglycan binding, heparin binding, hyaluronic acid binding, and polysaccharide binding, indicating overlapping ECM features and mechanisms associated with cartilage.

**Figure 9:**
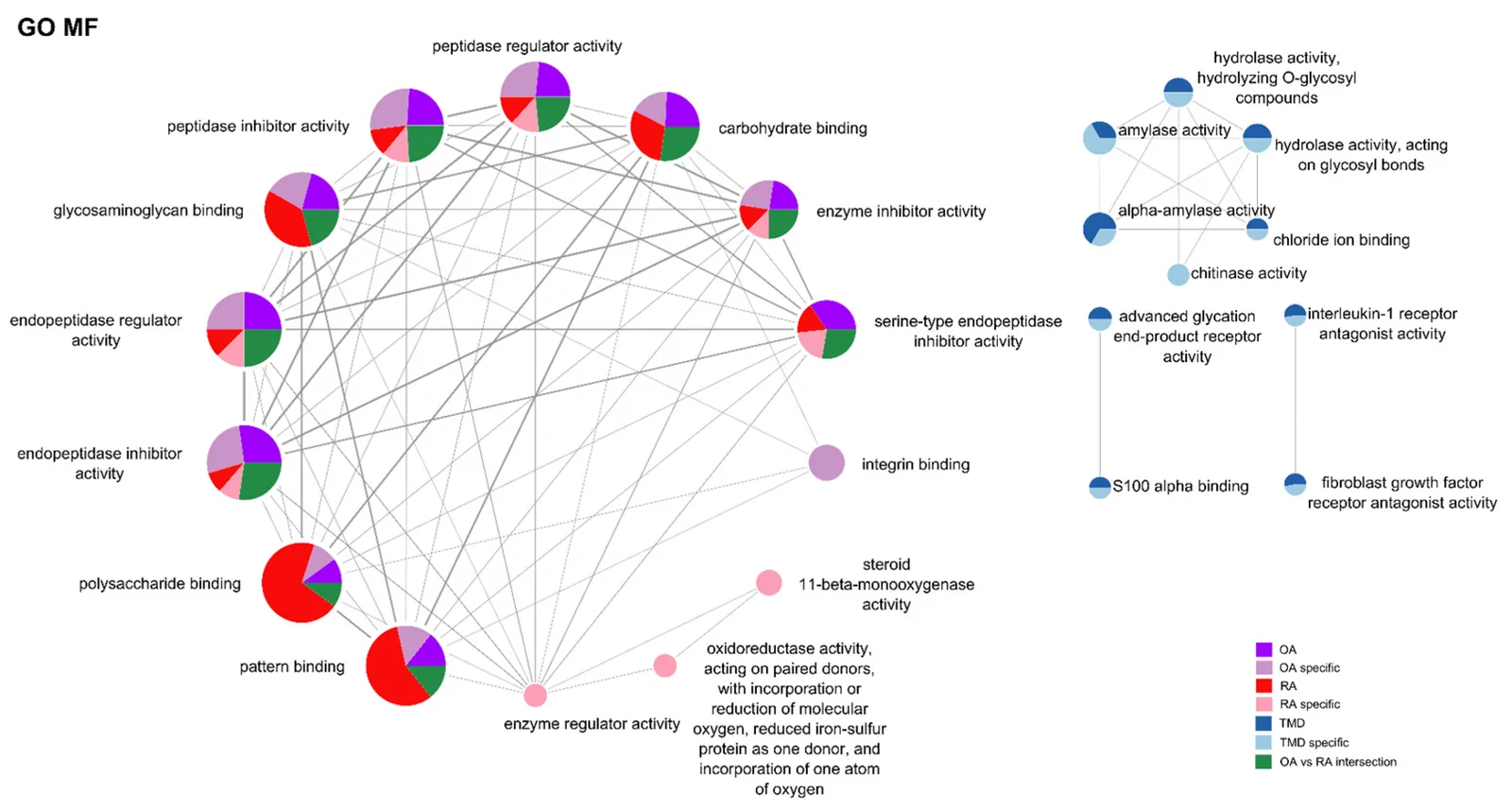
Gene Ontology Molecular Function (GO-MF) enrichment map network. Enrichment map network of the top ten enriched Gene Ontology Molecular Function (GO-MF) terms across OA, RA, TMD, disease-specific protein sets, and the OA–RA intersection. Nodes represent enriched GO terms and are scaled according to enrichment significance (−log10(FDR)). Pie-chart sectors show contributions from the individual disease groups. Edges connect GO terms that share genes, and the edge width corresponds to the number of shared genes. A highly interconnected core network centers on pattern binding, polysaccharide binding, glycosaminoglycan binding, endopeptidase inhibitor activity, and serine-type endopeptidase inhibitor activity. This enrichment pattern indicates common ECM interactions and regulation of proteolytic activity in OA and RA. OA-specific enrichment relates to integrin binding and extracellular matrix-associated functions, whereas proteins unique to the TMD dataset within this review are associated with amylase activity, hydrolase activity acting on glycosyl bonds, advanced glycation end-product receptor activity, and other salivary defense functions. The prominence of these functions is consistent with the salivary origin of the TMD dataset and therefore should not be interpreted as evidence of a disease-specific TMD functional signature.

**Figure 10:**
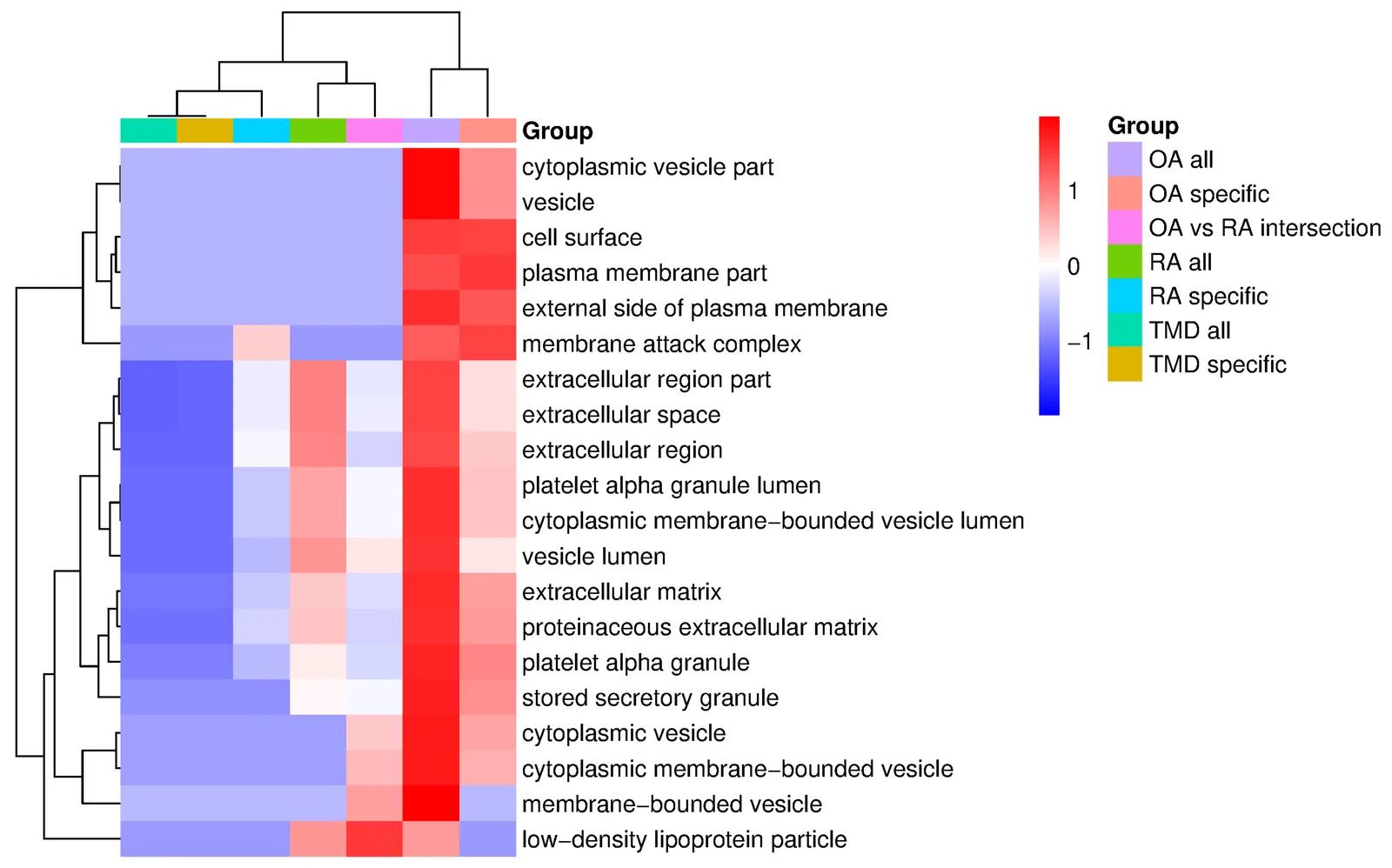
Gene Ontology Cellular Component (GO-CC) enrichment heatmap. Hierarchical clustering heatmap displaying the top 20 enriched Gene Ontology Cellular Component (GO-CC) terms across OA, RA, TMD, disease-specific protein sets, and the OA–RA intersection. Values indicate row-scaled −log10(FDR) enrichment scores (Z-score transformed). The most enriched proteins are mainly localized in extracellular compartments, such as the extracellular region, extracellular space, extracellular matrix, and proteinaceous extracellular matrix. RA proteins show strong enrichment in extracellular and vesicle-related components, while OA-specific proteins display a preference for ECM structures. TMD proteins are enriched in membrane and vesicular compartments, including the vesicle lumen, cytoplasmic vesicle, and membrane-bounded vesicle. Clustering reveals a shared extracellular functional compartment among all disease groups, highlighting the central role of secreted and matrix-associated proteins in the pathogenesis of OA, RA, and TMD.

**Figure 11:**
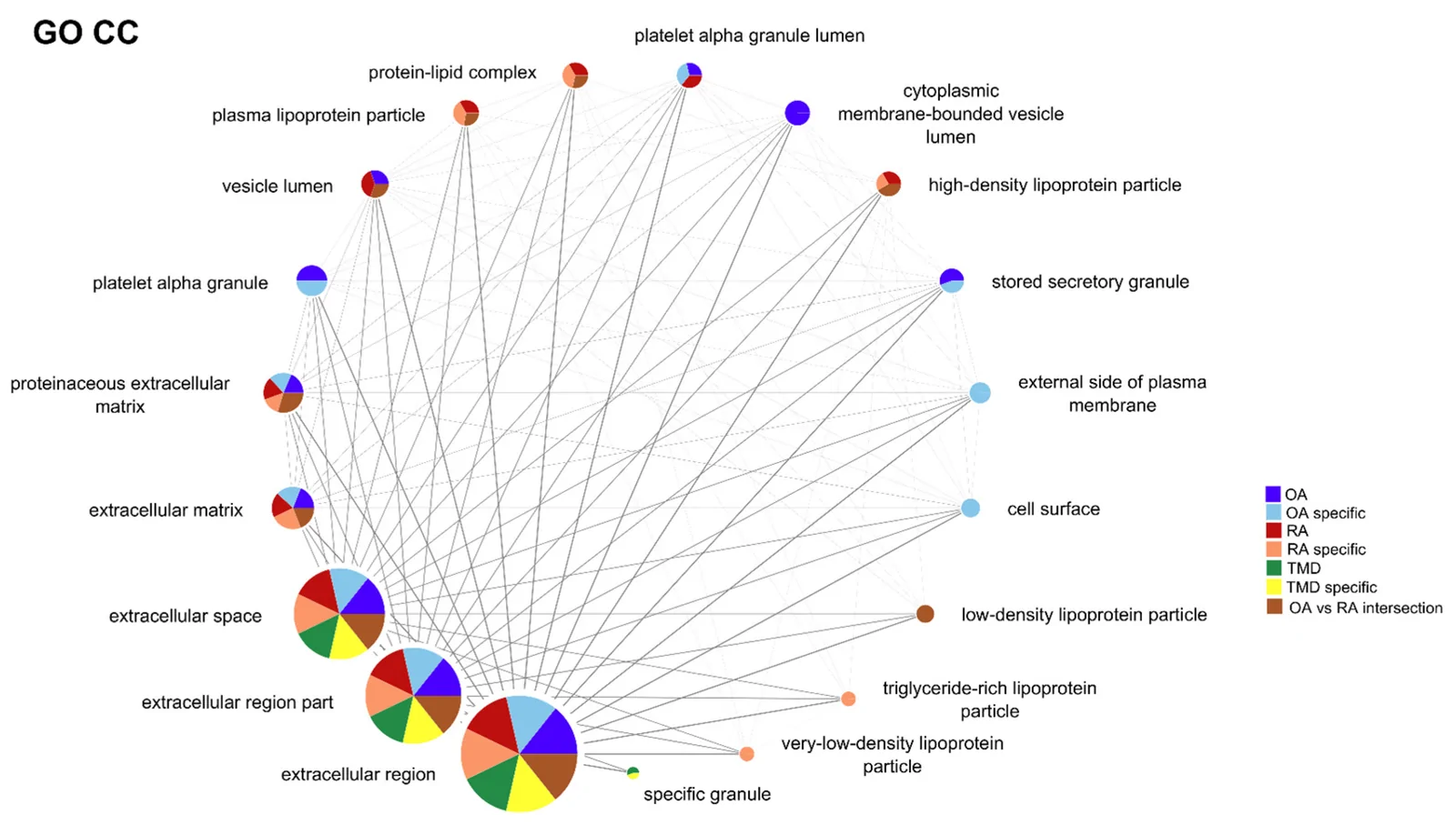
Gene Ontology Cellular Component (GO-CC) enrichment map network. Enrichment map network of the top ten enriched Gene Ontology Cellular Component (GO-CC) terms identified in OA, RA, TMD, disease-specific protein sets, and the OA–RA intersection. Nodes represent enriched cellular components and are sized according to enrichment significance (−log10(FDR)). Pie-chart sectors show the contribution of each disease group to the corresponding GO term. Edges connect GO terms that share common genes, and the edge width indicates how many genes are shared. The network is mainly dominated by a large extracellular cluster that includes extracellular region, extracellular region part, extracellular space, extracellular matrix, and proteinaceous extracellular matrix, showing that most proteins involved in disease are secreted or matrix associated. Other clusters involve vesicle-related components (vesicle lumen, platelet alpha granule lumen, stored secretory granule) and lipoprotein particles (low-density lipoprotein particle, high-density lipoprotein particle, and protein–lipid complex). The strong connectivity among the extracellular and secretory compartments reflects the role of extracellular signaling, matrix remodeling, and protein secretion in OA, RA, and TMD.

GO enrichment analysis was carried out separately for all disease-associated genes (OA all, RA all, TMD all), disease-specific genes (OA specific, RA specific, TMD specific), and genes shared between OA and RA (OA–RA intersection) (Figure 6, Figure 7, Figure 8, Figure 9, Figure 10 and Figure 11). GO enrichment analysis identified functional patterns associated with the OA, RA, and TMD datasets, focusing mainly on inflammatory processes and extracellular matrix-related functions. The complete OA gene set showed enrichment for biological processes linked to tissue injury, inflammation, and immune regulation. The most significant terms included response to wounding, acute inflammatory response, response to stress, and inflammatory response, along with activation of plasma proteins involved in acute inflammatory response and regulation of immune system processes. These enriched terms align with activation of innate immune and complement pathways. At the molecular-function (MF) level, OA genes were enriched for ECM-related interactions, including polysaccharide binding, glycosaminoglycan binding, carbohydrate binding, and endopeptidase inhibitor activity. This pattern largely corresponds to matrix glycoproteins such as ACAN, COMP, and PRG4. Cellular-component (CC) analysis indicated predominant localization in the extracellular region, extracellular space, and extracellular matrix. OA-specific genes maintained the inflammatory signature but displayed stronger enrichment for cell adhesion, biological adhesion, complement activation, and positive regulation of immune system processes. MF and CC enrichments also highlighted ECM organization and extracellular localization. The RA gene set showed enrichment for inflammatory and immune-related biological processes, such as acute inflammatory response, immune response, defense response, and response to wounding. MF enrichment indicated endopeptidase inhibitor, peptidase regulator, and serine-type endopeptidase inhibitor activities, mainly influenced by SERPIN family glycoproteins and acute-phase proteins that regulate extracellular proteolysis. CC analysis again revealed strong enrichment of extracellular compartments. RA-specific genes displayed a comparable pattern, characterized by immune activation, host defense, protease inhibition, and extracellular localization. Moreover, the smaller TMD gene set showed an enrichment pattern characterized by several salivary and innate immune functions. Enriched biological processes included digestion, immune response, defense response, inflammatory response, and positive regulation of NF-κB transcription factor activity. Key contributors were TLR4, AGER, IL1RN, and CRISP3, indicating involvement in innate immune signaling. The main molecular functions involved amylase activity, hydrolase activity acting on glycosyl bonds, and advanced glycation end-product receptor activity. These findings align with the presence of salivary proteins such as AMY1A, AMY2B, and AGER. CC analysis revealed enrichment in the extracellular region, extracellular space, and the lipopolysaccharide receptor complex. TMD-specific genes displayed similar enrichment patterns, mainly characterized by salivary enzyme activity, carbohydrate metabolism, and innate immune responses. Because the TMD dataset was derived predominantly from saliva, these enrichment differences cannot be interpreted as TMD-specific functional characteristics independently of biofluid composition. Genes shared between OA and RA showed enrichment profiles marked by inflammatory and ECM-related processes. The most notable biological processes included acute inflammatory response, response to wounding, inflammatory response, regulation of body fluid levels, and regulation of response to stimulus. MF enrichment indicated polysaccharide binding, pattern binding, glycosaminoglycan binding, and endopeptidase regulatory activities. Key contributors such as ACAN, COMP, PRG4, FN1, HRG, SERPINA1, and SERPIND1 connected cartilage matrix organization with the regulation of extracellular proteolysis. These shared proteins were mainly associated with the extracellular and matrix environment, highlighting overlapping pathways related to ECM organization, lubrication, complement regulation, and inflammatory signaling.

In summary, the enrichment findings revealed ECM remodeling and complement-mediated inflammatory processes in OA, immune and inflammatory pathways in RA, salivary enzyme and innate immune functions in TMD, and shared extracellular inflammatory processes within the OA and RA intersection.

### 3.7. Reactome Pathway Analysis

An overrepresentation analysis of Reactome pathways was conducted, including IntAct interactors to broaden the scope of the study. Reactome pathway analysis of proteins shared between OA and RA (Table 1) revealed seven significantly enriched pathways (FDR < 0.05), mainly linked to ECM remodeling, growth factor regulation, immune signaling, and platelet-driven inflammatory responses. The most significantly enriched pathways were Post-translational Protein Phosphorylation and Regulation of Insulin-like Growth Factor Transport and Uptake by IGF-Binding Proteins, both related to extensive extracellular protein processing and growth factor–related extracellular regulation. The notable enrichment of Platelet Degranulation and Responses to Elevated Platelet Cytosolic Ca^2+^ also suggested the involvement of platelet-derived inflammatory mediators. Among these enriched pathways, ECM Proteoglycans and Interleukin-4/Interleukin-13 Signaling were connected to cartilage matrix turnover, immune regulation, and ECM remodeling, representing key pathological mechanisms shared by OA and RA.

Reactome analysis of proteins shared by RA and TMD revealed (Table 2) that the main enriched pathways were linked to MAPK signaling, including Negative Feedback Regulation of MAPK Pathway, RAF/MAP Kinase Cascade, MAPK1/MAPK3 Signaling, and MAPK family signaling cascades. These were followed by FGFR signaling, which comprised Signaling by FGFR1-4, Negative Regulation of FGFR1-4 Signaling, and Spry Regulation of FGF Signaling. The enrichment pattern was largely dominated by growth factor–dependent signaling and MAPK-mediated pathways. These pathways were connected with FGFR–MAPK signaling, cellular proliferation, differentiation, and ECM remodeling. Altogether, they support a role for FGFR–MAPK signaling in inflammation, matrix remodeling, and cellular stress responses relevant to both RA and TMD. The enrichment of NCAM-related pathways also indicates the involvement of neuronal signaling and neurite outgrowth processes, suggesting a role for neuroinflammatory mechanisms responsible for pain sensation in RA and TMD.

Unlike the RA–TMD comparison, no Reactome pathways remained significant after multiple-testing correction in the OA–TMD dataset (Table 3). Although no pathways stayed significant following FDR correction, the top-ranked nominally enriched pathways were mainly linked to Toll-like receptor signaling, innate immune activation, pyroptosis, NF-κB signaling, and antigen processing, indicating inflammatory processes that might be shared between OA and TMD. A few pathways connected to cell–matrix interactions, such as Syndecan Interactions and Non-integrin Membrane–ECM Interactions, were also found among the top-ranked nominally enriched pathways. These pathways describe interactions between ECM proteoglycans and cell-surface receptors that influence adhesion, mechanotransduction, and inflammatory signaling.

In summary, Reactome enrichment analysis revealed recurring themes of inflammation, ECM remodeling, and disrupted tissue homeostasis across OA, RA, and TMD. OA−RA shared pathways were mainly linked to ECM organization and growth factor regulation, RA–TMD pathways were enriched in FGFR–MAPK and neuronal signaling, while OA–TMD displayed nominal enrichment of innate immune, TLR signaling, and pyroptosis-related pathways.

### 3.8. Glycan Pattern Analysis

Among the limited studies providing direct glycan-level information (Table 4), the reported glycosylation alterations in OA and RA predominantly involved O-glycans. Reported features included T antigen (Core 1 O-glycan), sialyl-T antigen, sialylated Core 1 structures, and truncated O-glycans. OA displayed the broadest variety of reported glycan structures, such as Tn antigen, undersialylated forms, and several sialylated O-glycan species linked mostly to lubricin (PRG4). In RA, the glycan changes were chiefly represented by Core 1 O-glycans of MMP3, sialyl-T structures, and α2,6-sialylated glycans. No distinct glycan structures were identified in the TMD studies included. Detailed structural information on glycans was available from only a few studies, mostly those involving PRG4 in OA and MMP3 in RA. Within this limited glycan-level evidence base, the reported alterations in OA and RA included truncated and sialylated O-glycan structures; however, the small number of studies precludes broader conclusions regarding disease-associated glycosylation patterns.

### 3.9. Risk-of-Bias Sensitivity Analysis

#### 3.9.1. Gene Ontology Analysis

After exclusion of the seven studies classified as having serious overall risk of bias, Gene Ontology (GO) overrepresentation analysis was repeated for the 82 OA-associated, 102 RA-associated, 9 TMD-associated, and 19 OA–RA shared proteins retained in the sensitivity dataset (Table 5).

In OA, the strongest biological-process signals shifted toward cell adhesion and biological adhesion (both FDR = 3.13 × 10^−11^), while response to wounding (FDR = 2.06 × 10^−9^), inflammatory response (FDR = 6.75 × 10^−5^), acute inflammatory response (FDR = 1.15 × 10^−3^), collagen metabolic process (FDR = 2.22 × 10^−5^), collagen catabolic process (FDR = 2.02 × 10^−3^), and extracellular matrix organization (FDR = 1.10 × 10^−2^) remained significantly enriched. OA molecular functions remained dominated by glycosaminoglycan binding (FDR = 1.10 × 10^−15^), polysaccharide and pattern binding (both FDR = 1.61 × 10^−15^), carbohydrate binding (FDR = 6.44 × 10^−13^), and endopeptidase inhibitory/regulatory activities, whereas cellular-component enrichment remained strongly extracellular, including extracellular region (FDR = 5.38 × 10^−37^), extracellular space (FDR = 1.58 × 10^−31^), and extracellular matrix (FDR = 3.80 × 10^−11^). The RA sensitivity set retained a pronounced inflammatory and protease-regulatory profile, with response to wounding (FDR = 1.02 × 10^−16^), acute inflammatory response (FDR = 6.52 × 10^−16^), inflammatory response (FDR = 5.89 × 10^−12^), defense response (FDR = 1.64 × 10^−9^), and complement activation (FDR = 9.73 × 10^−7^), together with endopeptidase inhibitor/regulator and serine-type endopeptidase inhibitor activities and strong extracellular localization. The TMD sensitivity set was characterized by digestion, immune and defense responses, inflammatory response, positive regulation of NF-κB transcription factor activity, amylase and glycosidase-related functions, and extracellular localization. In the reduced OA–RA intersection, regulation of response to stimulus (FDR = 1.31 × 10^−3^), acute inflammatory response (FDR = 1.52 × 10^−2^), response to wounding (FDR = 1.81 × 10^−2^), and inflammatory response (FDR = 1.81 × 10^−2^) remained significantly enriched. The shared molecular-function profile continued to include heparin, glycosaminoglycan, polysaccharide, and pattern binding, together with endopeptidase/peptidase inhibitory-regulatory activities, while the shared cellular-component profile remained dominated by extracellular region (FDR = 5.71 × 10^−16^), extracellular space (FDR = 1.17 × 10^−15^), and extracellular matrix (FDR = 2.69 × 10^−3^).

#### 3.9.2. Reactome Pathway Analysis

After exclusion of studies classified as having serious overall risk of bias, the OA–RA shared set contained 19 proteins and yielded five Reactome pathways that remained significant after false-discovery-rate correction (FDR < 0.05). The strongest enrichment was observed for Post-translational protein phosphorylation (FDR = 3.07 × 10^−6^; C4B, TF, IL6, FN1, APOB, and KNG1), followed by Regulation of Insulin-like Growth Factor (IGF) transport and uptake by Insulin-like Growth Factor Binding Proteins (IGFBPs) (FDR = 1.61 × 10^−5^; C4B, TF, IL6, FN1, IGFBP6, APOB, and KNG1). Interleukin-4 and Interleukin-13 signaling (FDR = 0.00491; IL6, MMP3, and FN1), LDL remodeling (FDR = 0.0137; APOF and APOB), and Platelet degranulation (FDR = 0.0311; TF, FN1, HRG, and KNG1) were also retained. By contrast, Degradation of the extracellular matrix, although nominally enriched (*p* = 0.0106; ACAN, MMP3, and FN1), did not remain significant after multiple-testing correction (FDR = 0.293). No proteins were shared between OA and TMD in the sensitivity dataset; consequently, no OA–TMD Reactome pathway analysis was applicable. The RA–TMD overlap was reduced to a single protein, AZGP1. Twenty Reactome terms had FDR < 0.05 in the ReactomeFI output; however, only Miscellaneous transport and binding events directly contained AZGP1 as a submitted pathway entity (FDR = 0.0245). The remaining significant terms, including NCAM1 interactions, NCAM signaling for neurite out-growth, negative regulation of the MAPK pathway, Spry regulation of FGF signaling, signaling by FGFR1–4, RAF/MAP kinase cascade, and MAPK1/MAPK3 signaling, were generated through AZGP1-linked interactors rather than direct membership of AZGP1 in those pathways.

#### 3.9.3. Sensitivity Network Topology

After exclusion of the seven studies classified as having serious overall risk of bias, the reconstructed disease–protein network contained 176 nodes, comprising 3 disease nodes and 173 unique protein nodes, connected by 193 disease–protein edges. Compared with the primary network (202 nodes and 230 edges), network density increased slightly from 0.011 to 0.013, while network heterogeneity decreased from 4.765 to 4.428 and centralization remained similar (0.566 vs. 0.577). The characteristic path length increased from 2.79 to 2.93 and the network diameter increased from 4 to 6, reflecting loss of the direct OA–TMD protein overlap after risk-of-bias filtering. The OA–RA intersection decreased from 29 to 19 proteins, whereas AZGP1 remained the only protein shared between RA and TMD. Consequently, AZGP1 showed increased betweenness centrality (0.098 versus 0.037 in the primary network), while TLR4 no longer occupied an OA–TMD cross-disease position. MMP3 remained the most recurrently supported protein, with sensitivity weighted node strength of 6 compared with 7 in the primary analysis. Overall, the sensitivity network retained a prominent OA–RA component but showed reduced cross-disease connectivity involving TMD.

Sensitivity edge weight was recalculated from distinct retained studies (Supplementary Table S3).

## 4. Discussion

### 4.1. Network Topology and Mechanistic Implications

Within this literature-derived association network, the observed topology primarily describes patterns of recurrence and cross-disease reporting rather than molecular interaction or regulatory organization. Proteins with higher weighted degree were supported by a greater number of reported disease–protein associations, whereas proteins with higher degree or betweenness occurred across more than one disease category and therefore occupied cross-disease positions within the evidence structure. Matrix metalloproteinases and extracellular matrix-associated glycoproteins were prominent among these recurrently reported proteins. Their established roles in collagen degradation, tissue remodeling, and inflammatory processes are supported by independent biological studies [30,31], but these functions cannot be inferred from network centrality itself. Similarly, the relatively high betweenness values of TLR4 and AZGP1 indicate that these proteins connect disease categories within the literature-derived graph; they do not demonstrate molecular bridging, causal regulatory activity, or therapeutic relevance. Accordingly, the network topology is used here to summarize the structure of the available evidence and to identify candidates for subsequent mechanistic and clinical validation rather than to infer biological hub or therapeutic-target status.

### 4.2. Functional Interpretation of Enrichment Analyses

The functional enrichment results reinforce the prominence of ECM turnover across the integrated molecular dataset. The enrichment of collagen catabolic and metabolic processes, along with metallopeptidase activity, indicates the predominance of MMP-mediated proteolysis in the molecular environment. This finding is in accordance with the well-established role of MMPs in cartilage degradation and tissue remodeling across inflammatory and degenerative joint diseases [67,68]. The enrichment of extracellular region and ECM cellular components further confirms that the identified molecular processes are limited to the extracellular compartment, consistent with the biological context of synovial fluid and salivary biomarkers [21]. In addition, the presence of protease inhibitors, including TIMP1 and SERPIN family members, highlights a carefully regulated proteolytic environment in which matrix compartment degradation is balanced by inhibitory mechanisms [68]. Disruption of this balance is a defining feature of chronic inflammatory conditions and contributes to ongoing tissue damage. The enrichment analysis supports the idea that ECM degradation is controlled by coordinated proteolytic networks rather than by isolated molecular events.

### 4.3. Impact of Study Heterogeneity and Bias on Interpretation

The interpretation of the results should be viewed in light of the methodological limitations of the included studies. Comparator definitions varied among the included studies and ranged from healthy reference groups to disease-specific comparators. This heterogeneity limits direct comparison of biomarker contrasts across studies and was therefore considered in the risk-of-bias assessment. The 2016 lower-date restriction may have excluded earlier studies containing relevant protein- or glycan-level observations. Consequently, the review should be interpreted as a synthesis of contemporary evidence rather than an exhaustive historical survey of glycoprotein and glycosylation research. Most of these studies were judged to have a moderate to serious risk of bias, mainly because of confounding, participant selection, and selective reporting. Such factors may influence both the magnitude and specificity of the observed biomarker associations, especially for immune-related pathways that respond to systemic variability. The limited number of studies also highlights the lack of investigations integrating proteomic and glycoproteomic analyses across OA, RA, and TMD. A potential limitation of this study lies in the recognized heterogeneity of OA, RA, and TMD as biological conditions. Moreover, synovial fluid and saliva can be regarded as heterogeneous biofluids because of the basic differences in their matrices. Therefore, future comparative studies should include both synovial fluid and saliva samples for each disease to enable direct comparisons across biological matrices. This point also applies to the few glycomics studies that have described glycan structures.

Despite this methodological heterogeneity, extracellular matrix remodeling and regulation of extracellular proteolysis remained recurrent themes across the integrated evidence Their recurrence across studies using different analytical platforms and populations supports their relevance at the process level, although individual proteins and more specific pathway assignments were less stable in the risk-of-bias sensitivity analysis [67,68]. Immune-related findings showed greater variability and should therefore be interpreted more cautiously [69]. Together with the limited availability of glycan-level data, these considerations indicate that the present molecular patterns are hypothesis-generating and require validation using quantitative, site-specific glycoproteomic approaches [12,20].

Because the disease-level network collapses observations across biofluids, cross-disease centrality may partly reflect the integration of matrix-specific biomarker sets rather than shared in vivo molecular connectivity; these topological measures should therefore be interpreted only as properties of the literature-derived evidence structure.

Although the review concentrated on glycoprotein-associated biomarkers, most existing studies presented protein-level rather than glycan-level information. As a result, the identified molecular signatures mainly represent proteomic alterations involving glycoproteins. Future studies combining site-specific glycoproteomics and glycomics will be needed to clarify the role of glycan remodeling in OA, RA, and TMD pathogenesis.

#### 4.3.1. Limited Glycan-Level Evidence and the Glycosylation Knowledge Gap

Although most included studies did not directly characterize glycosylation, the recurrent identification of glycoproteins such as PRG4, COMP, and MMP3 provides a biologically informed basis for prioritizing candidates for future site-specific glycoproteomic investigation.

The paucity of glycan-level information is both a limitation of the present evidence synthesis and a clearly defined research gap. The current evidence base is predominantly protein-centric, and the functional relevance of glycosylation in these conditions remains largely uncharacterized.

#### 4.3.2. Guidance for Future Glycoproteomic Study Design

Based on the findings of this review, several methodological considerations may inform the design of future glycoproteomic studies. Targeted site-specific glycoproteomics. Future investigations may benefit from moving beyond broad discovery-based proteomics toward targeted mass spectrometry approaches, such as parallel reaction monitoring or selected reaction monitoring. These methods can characterize site-specific glycosylation of recurrently reported candidate proteins identified in this review, including PRG4, MMP3, SERPINA1, and COMP. Such approaches would allow for the mapping of glycan occupancy and microheterogeneity at individual glycosylation sites, which is a prerequisite for functional correlation. Multi-modal analytical strategies. A single analytical technique is unlikely to fully capture glycoproteomic complexity. A tiered approach may be considered: (a) quantitative proteomics (e.g., DIA or TMT) to establish baseline protein abundance; (b) site-specific glycoproteomics (using EThcD or HCD fragmentation) to map glycan structures and their attachment sites; and (c) complementary glycomics (released glycan analysis via LC-MS/MS) to capture the global glycan repertoire of the biofluid, providing context for protein-specific findings. Standardized biofluid collection and processing. Variability in saliva composition—influenced by oral microbiota, flow rate, and dietary factors—can complicate the interpretation of systemic signals. Standardized collection protocols, including fasting and unstimulated whole saliva collection, along with the use of protease inhibitors and defined quality control metrics for storage and processing, would improve comparability across studies. Paired biofluid studies. To distinguish biofluid-specific effects from disease-specific mechanisms, future research could include paired synovial fluid and saliva samples from the same patient cohorts across OA, RA, and TMD. This design would enable intra-individual correlation between local joint pathology and systemic or non-invasive signatures, thereby facilitating an assessment of the translational potential of salivary glycobiomarkers. Longitudinal cohort designs. Cross-sectional studies are limited in their capacity to distinguish disease progression from static pathology. Glycoproteomic investigations nested within prospective, well-phenotyped longitudinal cohorts would allow for tracking of glycoform changes over time in relation to disease progression, treatment response, or remission. Implementation of these approaches toward hypothesis-driven, site-specific glycoproteomic techniques would enable the field to move beyond identifying protein presence and toward elucidating the functional contributions of glycosylation in joint pathology.

### 4.4. Reactome Analysis

#### 4.4.1. OA–RA Shared Proteins

The OA–RA Reactome profile converged on extracellular protein processing, regulation of growth-factor bioavailability, platelet-associated responses, and cytokine signaling. Rather than representing independent mechanisms, these pathways collectively support an extracellular environment in which matrix turnover, inflammatory signaling, and tissue repair are closely coupled. The presence of TLR4 within the shared protein set is consistent with established links between innate immune activation and tissue remodeling; however, its biological role cannot be inferred from its position in the literature-derived network alone [70].

#### 4.4.2. RA–TMD Shared Proteins

The primary RA–TMD Reactome analysis suggested possible involvement of growth-factor/MAPK and NCAM-associated signaling. These pathways are biologically relevant to inflammatory signaling, tissue remodeling, and neuroimmune processes; however, their interpretation requires particular caution in the present dataset. The RA–TMD overlap was very small and was further reduced to AZGP1 alone after exclusion of studies with serious risk of bias. Moreover, most pathway assignments retained in the sensitivity analysis were generated through AZGP1-linked interactors rather than direct pathway membership. Accordingly, the FGFR–MAPK and NCAM findings should be regarded as exploratory hypotheses rather than evidence of a robust molecular program shared by RA and TMD. The marked difference in biofluid composition between the RA and TMD evidence bases further limits disease-specific interpretation.

#### 4.4.3. OA–TMD Shared Proteins

The OA–TMD comparison provides considerably weaker evidence than the OA–RA comparison because no Reactome pathway remained significant after correction for multiple testing. The nominal enrichment of pathways related to innate immune signaling and matrix–cell interactions is biologically compatible with established roles of TLR4/NF-κB signaling in inflammatory and mechanically stressed joint tissues [71,72,73,74,75,76,77,78,79], but it should not be regarded as evidence of a disease-specific shared pathway. This interpretation is reinforced by the loss of the OA–TMD protein overlap in the risk-of-bias sensitivity analysis and by the marked difference in biofluid composition between the OA and TMD datasets.

#### 4.4.4. Extracellular Matrix Remodeling and Regulation of Extracellular Proteolysis as Shared Disease Mechanisms

Taken together, the network, GO, and Reactome analyses converge on extracellular matrix remodeling and regulation of extracellular proteolysis as the principal process-level features shared by OA and RA. This convergence involves matrix glycoproteins, metalloproteinases, protease inhibitors, complement-associated proteins, and inflammatory mediators, indicating that tissue remodeling and inflammatory regulation are closely interconnected rather than independent processes. Importantly, the sensitivity analysis indicates that this conclusion is more robust at the broad functional level than at the level of individual proteins or specific pathways. Thus, recurrent proteins such as PRG4, COMP, FN1, ACAN, and SERPINA1 should be considered candidates that contribute to the observed molecular pattern rather than universal cross-disease biomarkers.

### 4.5. Risk-of-Bias Sensitivity Analysis

Because each independent study contributes to the literature-derived evidence structure irrespective of its risk-of-bias classification, proteins reported predominantly in methodologically weaker studies can influence both network prominence and downstream enrichment. The sensitivity analysis excluding serious-risk studies was therefore used to assess whether the principal findings depended on these studies. Accordingly, findings that were retained in both analyses can be considered more robust within the limits of the available literature, whereas proteins or pathways lost after exclusion of serious-risk studies should be regarded as less stable and hypothesis-generating.

Comparison of the primary and sensitivity GO analyses showed that the major functional conclusions were generally preserved, although robustness differed among disease groups. OA retained extracellular-matrix, adhesion, inflammatory, and remodeling signals, whereas some complement-related components were less stable. The RA functional profile was comparatively robust, while the unchanged TMD protein set reproduced its salivary enzymatic and innate-immune profile without resolving the confounding effect of biofluid composition. Most importantly, despite contraction of the OA–RA shared protein set, the broader extracellular inflammatory and proteolytic-remodeling signature remained evident, indicating greater robustness at the process level than at the level of individual proteins.

The Reactome sensitivity analysis similarly narrowed the interpretation of the OA–RA overlap. Although inflammatory and extracellular-regulatory pathways remained supported, ECM degradation no longer survived FDR correction, coinciding with the loss of COMP, PRG4, SERPINA1, and SERPIND1 from the shared set. Thus, the broad extracellular/remodeling signal was robust, whereas the specific ECM-degradation and protease-regulatory components were more dependent on studies with serious methodological limitations. Evidence for shared RA–TMD and OA–TMD mechanisms was substantially weaker after risk-of-bias filtering.

## 5. Conclusions

This systematic review provides an integrative analysis of glycoprotein and proteomic biomarkers in OA, RA, and TMD, with emphasis on synovial fluid and saliva as biologically and clinically relevant matrices. Comparisons involving TMD remain exploratory because the available TMD evidence was limited and predominantly saliva-based, whereas OA and much of the RA evidence originated from synovial fluid; consequently, the present analysis cannot distinguish TMD-specific molecular differences from biofluid-related effects. Network topology, Gene Ontology enrichment, and pathway analyses identified extracellular matrix remodeling, regulation of extracellular proteolysis, and the structural and integrative roles of glycoproteins, including PRG4, FN1, COMP, and ACAN. However, sensitivity analysis shows that PRG4 and COMP are lost from the OA–RA shared set and Reactome ECM degradation no longer survives FDR correction. The broad extracellular/remodeling signal was robust while specific ECM-degradation and protein-level components were more study-quality-sensitive.

The integrative analyses in this review primarily identify protein-level associations involving experimentally annotated glycoproteins. They support recurrent involvement of extracellular matrix remodeling and extracellular proteolytic regulation in OA and RA, but they do not establish that altered glycosylation drives these processes. Direct glycan-level evidence was available in only 5 of 38 included studies and was restricted to a small number of proteins and glycan features. Thus, the contribution of disease-associated glycosylation remains insufficiently characterized. The proteins recurrently identified in the present synthesis provide candidates for future site-specific glycoproteomic investigation rather than validated glycoform-specific biomarkers.

A key finding of this review is the limited availability of direct glycan-level data in the existing literature. The current evidence base is predominantly protein-centric, and the functional roles of glycosylation—a dynamic post-translational modification influencing protein stability, immune recognition, and intercellular communication—remain largely inferred rather than empirically defined. The literature-derived protein networks identified recurrently reported ECM proteins and inflammatory mediators that may be prioritized as candidates for future site-specific glycoproteomic studies. Future research would benefit from standardized biofluid collection protocols, paired synovial fluid-saliva sampling within longitudinal cohorts, and the integration of complementary mass spectrometry-based workflows to resolve glycan microheterogeneity. The complementary analysis of synovial fluid and saliva offers considerable potential for translating proximal joint pathology into non-invasive diagnostic and monitoring strategies. Realizing this potential will require rigorous characterization of the molecular complexity of glycoproteins, particularly their glycan modifications. The absence of such data in the current literature defines an important priority for future biomarker research in inflammatory joint diseases.

## 6. Future Perspectives

The findings of this review highlight the need to move beyond predominantly protein-focused analyses toward a broader characterization of glycoproteins and their glycan structures in OA, RA, and TMD. While extracellular environment dynamics and proteolytic activity are both recognized as key mechanisms of these diseases, glycoproteins—and in particular, their glycosylation status—remain a relatively unexplored yet highly informative aspect of molecular regulation. Future research should emphasize the integration of glycoproteomic and glycomic approaches capable of resolving site-specific glycosylation patterns and protein-specific glycoforms. Glycans act as dynamic regulators that influence protein stability, receptor binding, and immune recognition. A systematic characterization of glycan features, including sialylation, fucosylation, and truncated O-glycans, will be essential for understanding how glycoproteins shape joint microenvironment dynamics. Special attention should be directed toward key glycoproteins identified in this review, such as proteoglycan 4 (PRG4), matrix metalloproteinases, and protease inhibitors, whose functional properties may be substantially modified by glycosylation. Defining the glycoform-specific behavior of these molecules could offer valuable insight into disease progression and reveal regulatory mechanisms that cannot be detected through protein abundance measurements alone. From a methodological standpoint, future studies should employ multicomponent analytical strategies that integrate quantitative proteomics, site-specific glycoproteomics, and targeted glycan profiling within well-defined clinical cohorts. Standardizing sample collection and processing, particularly for saliva, will be vital to minimize variability and support cross-study comparisons. The combined use of synovial fluid and saliva represents a promising approach for linking local joint pathology with non-invasive biomarker detection. In this regard, glycoproteins and their glycans are increasingly recognized as important translational targets. From a clinical standpoint, developing biomarker panels informed by glycoprotein and glycan data is a logical next step. These panels could improve disease stratification, allow earlier detection of pathological changes, and enable more precise monitoring of therapeutic responses. Future studies using matched biofluids and appropriately designed comparative cohorts should determine whether glycosylation patterns can provide reproducible disease-specific signatures capable of differentiating OA, RA, and TMD. Recent progress in high-resolution mass spectrometry, glycan enrichment methods, and computational network analysis is expected to greatly expand both the analytical depth and interpretability of glycoproteomic data. Incorporating these technologies within systems biology frameworks will support a more integrated understanding of extracellular molecular networks and their regulation through glycosylation. Focusing on the structural and functional roles of glycoproteins and glycans is likely to advance biomarker discovery and deepen the mechanistic understanding of OA, RA, and TMD.

## Figures and Tables

**Table 1:** Significantly enriched Reactome pathways of proteins common to OA and RA (FDR < 0.05).

Pathway Identifier	Pathway Name	Entity Ratio	Entity *p* Value	Entity FDR
R-HSA-8957275	Post-translational Protein Phosphorylation	0.006732967	6.16 × 10^−11^	2.71 × 10^−8^
R-HSA-381426	Regulation of Insulin-like Growth Factor (IGF) Transport and Uptake by Insulin-like Growth Factor Binding Proteins (IGFBPs)	0.007844833	4.48 × 10^−9^	9.81 × 10^−7^
R-HSA-114608	Platelet Degranulation	0.008771388	1.42 × 10^−5^	2.08 × 10^−3^
R-HSA-76005	Response to Elevated Platelet Cytosolic Ca2^+^	0.00920378	6.89 × 10^−5^	7.51 × 10^−3^
R-HSA-8964041	LDL Remodeling	0.000617703	3.29 × 10^−4^	0.027264806
R-HSA-6785807	Interleukin-4 and Interleukin-13 Signaling	0.013033541	3.73 × 10^−4^	0.027264806
R-HSA-3000178	ECM Proteoglycans	0.004879857	5.35 × 10^−4^	0.033165102

**Table 2:** Significantly enriched Reactome pathways of proteins common to RA and TMD (FDR < 0.05).

Pathway Identifier	Pathway Name	Entity Ratio	Entity *p* Value	Entity FDR
R-HSA-419037	NCAM1 Interactions	0.003460778	0.00366424	0.024492551
R-HSA-5674499	Negative Feedback Regulation of MAPK Pathway	0.000494397	0.00877489	0.024492551
R-HSA-5223345	Miscellaneous Transport and Binding Events	0.002389585	0.01007666	0.024492551
R-HSA-375165	NCAM Signaling for Neurite Out-growth	0.005273566	0.011715925	0.024492551
R-HSA-5675221	Negative Regulation of MAPK Pathway	0.003625577	0.013548045	0.024492551
R-HSA-1295596	Spry Regulation of FGF Signaling	0.001318392	0.014464105	0.024492551
R-HSA-5654733	Negative Regulation of FGFR4 Signaling	0.002554384	0.015090883	0.024492551
R-HSA-5654732	Negative Regulation of FGFR3 Signaling	0.002471984	0.015090883	0.024492551
R-HSA-5654727	Negative Regulation of FGFR2 Signaling	0.00304878	0.015428379	0.024492551
R-HSA-5654726	Negative Regulation of FGFR1 Signaling	0.002883982	0.015524806	0.024492551
R-HSA-9834899	Specification of the Neural Plate Border	0.001400791	0.017694422	0.024492551
R-HSA-5654741	Signaling by FGFR3	0.003543177	0.024396124	0.024492551
R-HSA-5654743	Signaling by FGFR4	0.003543177	0.024492551	0.024492551
R-HSA-5654736	Signaling by FGFR1	0.00461437	0.024926474	0.024926474
R-HSA-5673001	RAF/MAP Kinase Cascade	0.022989453	0.027963936	0.027963936
R-HSA-5654738	Signaling by FGFR2	0.006427159	0.032254954	0.032254954
R-HSA-190236	Signaling by FGFR	0.007992749	0.033604937	0.033604937
R-HSA-5684996	MAPK1/MAPK3 Signaling	0.023566249	0.034472783	0.034472783
R-HSA-9758941	Gastrulation	0.009475939	0.043970879	0.043970879

**Table 3:** Top Reactome pathways of proteins common to OA and TMD (nominal *p* < 0.05; no pathways remained significant after FDR correction).

Pathway Identifier	Pathway Name	Entity Ratio	Entity *p* Value	Entity FDR
R-HSA-9707616	Heme Signaling	0.003872775	0.001202098	0.063372423
R-HSA-5603041	IRAK4 Deficiency (TLR2/4)	0.001647989	0.002890032	0.063372423
R-HSA-5602498	MyD88 Deficiency (TLR2/4)	0.00156559	0.005630401	0.063372423
R-HSA-140534	Caspase Activation via Death Receptors in the Presence of Ligand	0.00156559	0.005918568	0.063372423
R-HSA-937041	IKK Complex Recruitment Mediated by RIP1	0.001895188	0.006638743	0.063372423
R-HSA-5686938	Regulation of TLR by Endogenous Ligand	0.001812788	0.006782736	0.063372423
R-HSA-2562578	TRIF-mediated Programmed Cell Death	0.000741595	0.008940965	0.063372423
R-HSA-3134963	DEx/H-box Helicases Activate Type I IFN and Inflammatory Cytokines Production	0.005767963	0.010808899	0.063372423
R-HSA-9820960	Respiratory Syncytial Virus (RSV) Attachment and Entry	0.002554384	0.012100708	0.063372423
R-HSA-975163	IRAK2 Mediated Activation of TAK1 Complex upon TLR7/8 or 9 Stimulation	0.001235992	0.012531061	0.063372423
R-HSA-937072	TRAF6-mediated Induction of TAK1 Complex within TLR4 Complex	0.001318392	0.012674485	0.063372423
R-HSA-5620971	Pyroptosis	0.002224786	0.014824167	0.071885545
R-HSA-9702518	STAT5 Activation Downstream of FLT3 ITD Mutants	0.000823994	0.017971386	0.071885545
R-HSA-5602358	Diseases Associated with the TLR Signaling Cascade	0.002883982	0.027798721	0.088928628
R-HSA-5260271	Diseases of Immune System	0.003295979	0.028933862	0.088928628
R-HSA-5357769	Caspase Activation via Extrinsic Apoptotic Signalling Pathway	0.002389585	0.029642876	0.088928628
R-HSA-9824878	Regulation of TBK1, IKKε (IKBKE)-mediated Activation of IRF3, IRF7	0.001153593	0.030209839	0.090422326
R-HSA-9703465	Signaling by FLT3 Fusion Proteins	0.001647989	0.031484698	0.090422326
R-HSA-3000170	Syndecan Interactions	0.002224786	0.031909403	0.090422326
R-HSA-1236974	ER-Phagosome Pathway	0.007003955	0.043469973	0.090422326
R-HSA-1236975	Antigen Processing-Cross Presentation	0.008322347	0.045714909	0.090422326
R-HSA-3000171	Non-integrin Membrane–ECM Interactions	0.006591958	0.048516136	0.090422326

**Table 4:** Glycan pattern analysis.

Glycan Class	Definition /Criterion	OA Evidence	RA Evidence	TMD Evidence	OA Proteins	RA Proteins
O-glycans	Any reported O-glycan structure or O-glycan cluster	HexNAc(1); HexNAc(1)Hex(1); HexNAc(1)Hex(1)NeuAc(1); HexNAc(1)Hex(1)NeuAc(2); HexNAc(2); HexNAc(2)Hex(1); HexNAc(2)Hex(2)NeuAc(1); HexNAc(2)Hex(2)NeuAc(2); Sialyl-T antigen; Sialylated core 1 O-glycans; T antigen/Core 1 (Galβ1-3GalNAcα1-Ser/Thr); Tn antigen (GalNAcα1-Ser/Thr); Truncated/undersialylated O-glycans	ACG/Jacalin ratio; O-glycan cluster on MMP3; Sialyl-T antigen; T antigen (Core 1 O-glycan); α2,6-sialylated O-glycan	NR	PRG4	MMP3
Tn antigen	GalNAcα1-Ser/Thr or HexNAc(1)-type truncated O-glycan	HexNAc(1); Tn antigen (GalNAcα1-Ser/Thr)	NR	NR	PRG4	
T antigen	Core 1 Galβ1-3GalNAcα1-Ser/Thr	T antigen/Core 1 (Galβ1-3GalNAcα1-Ser/Thr)	T antigen (Core 1 O-glycan)	NR	PRG4	MMP3
Sialyl-T	Sialylated T antigen/sialylated core 1	Sialyl-T antigen	Sialyl-T antigen	NR	PRG4	MMP3
Sialylated glycans	Any NeuAc-containing or linkage-defined sialylated glycan	HexNAc(1)Hex(1)NeuAc(1); HexNAc(1)Hex(1)NeuAc(2); HexNAc(2)Hex(2)NeuAc(1); HexNAc(2)Hex(2)NeuAc(2); Sialyl-T antigen; Sialylated core 1 O-glycans; Truncated/undersialylated O-glycans	Sialyl-T antigen; α2,6-sialylated O-glycan	NR	PRG4	MMP3
α2,6-sialylated	Explicit α2,6-sialylated O-glycan	NR	α2,6-sialylated O-glycan	NR		MMP3
Sialylated Core 1	Sialylated core 1 O-glycan/Sialyl-T	Sialyl-T antigen; Sialylated core 1 O-glycans	Sialyl-T antigen	NR	PRG4	MMP3
Truncated glycans	Tn, T antigen, or explicit truncated/undersialylated O-glycans	HexNAc(1); T antigen/Core 1 (Galβ1-3GalNAcα1-Ser/Thr); Tn antigen (GalNAcα1-Ser/Thr); Truncated/undersialylated O-glycans	T antigen (Core 1 O-glycan)	NR	PRG4	MMP3
Tn	Same as Tn antigen	HexNAc(1); Tn antigen (GalNAcα1-Ser/Thr)	NR	NR	PRG4	
T antigen (truncated)	T antigen/Core 1 treated as truncated/less-extended O-glycan	T antigen/Core 1 (Galβ1-3GalNAcα1-Ser/Thr)	T antigen (Core 1 O-glycan)	NR	PRG4	MMP3
Undersialylated structures	Explicit undersialylated structures; T/Tn as supportive evidence	Truncated/undersialylated O-glycans	NR	NR	PRG4	

**Table 5:** Risk-of-bias sensitivity table.

Disease	Primary Proteins	Sensitivity Proteins	Retained	Lost	Retention
OA	115	82	82	33	71.3%
RA	106	102	102	4	96.2%
TMD	9	9	9	0	100%

## Data Availability

Data is contained within the article.

## Supplementary Materials

The following supporting information can be downloaded at: https://www.mdpi.com/article/10.3390/ijms27198764/s1.
