# Supplementary material for: Extracellular Matrix Remodeling and Glycoprotein Biomarkers Across Osteoarthritis, Rheumatoid Arthritis, and Temporomandibular Disorders: A Systematic Review and Integrative Analysis
Source: Int J Mol Sci. 2026 Sep 30;27(19):8764. doi: 10.3390/ijms27198764 (PMC13664865; doi:10.3390/ijms27198764)
Supplement: Supplementary file 1 [file ijms-27-08764-s001.zip › Table S1.pdf]

Table S1. Characteristics of included studies.

Reference	Condition	Saliva/<br>synovial fluid	Method	Glycoprotein markers	Glycans	Controls	DOI
Afshari et al., 2025	OA	synovial fluid	glycomics + glycoproteomics MS (StcE enrichment / mucinase / trypsin); FILA	lubricin (PRG4)	Core 1 O-glycan (T antigen); sialyl-T antigen (mono- and multi-sialylated); extended/branched O-glycans; poly-LacNAc structures; possible fucosylated O-glycans	Healthy plasma controls; no healthy SF controls.	10.1016/j.mcpro.2025.100923
Ali et al., 2022	early OA; late OA	synovial fluid	DIA mass-spectrometry proteomics; co-expression/protein-interplay analysis	early OA showed more increased proteins vs controls than late OA; catabolic activity increased in both early and late OA; altered protein-protein interplay.	No glycan structures reported	Deceased donor synovial fluid controls without knee OA.	10.1016/j.mcpro.2022.100200
Arvikar et al., 2021	RA	saliva + synovial fluid	multiplex immunoassay + Pg IgG/DNA assays	MMP1, MMP3, MMP8, MMP9,inflammatory markers (CXCL8, IL10, IL17A) vs antibodies (Pg IgG)	No glycan structures reported	Healthy controls and non-RA chronic periodontitis controls.	10.1002/JPER.20-0051

Äyräväinen et al., 2018	RA	saliva	salivary/serum biomarker assay	MMP8, TIMP1, inflammatory markers (IL6)	No glycan structures reported	Healthy controls.	10.1080/07853890.2018.1468922
Barreto et al., 2019	OA	synovial fluid	synovial-fluid LUM measurement + TLR4/NFκB/cartilage/macrophage assays	lumican	No glycan structures reported	Healthy synovial fluid controls.	10.1016/j.joca.2019.10.011
Huang et al., 2022	OA	synovial fluid	lectin/glycosylation analysis of lubricin; cytokine assays; synoviocyte stimulation	lubricin (PRG4); interleukin-8, C-C motif chemokine 3, vascular endothelial growth factor Aassociation with PRG4glycoforms	Tn antigen (GalNAcα1-Ser/Thr), T antigen (Galβ1-3GalNAcα1-Ser/Thr), sialylated and nonsialylated T-related O-glycans; reduced sialylation	Healthy plasma controls and non-OA cadaveric SF controls.	10.3389/fmolb.2022.942406
Huang Y et al., 2022	RA, axSpA, gout vs OA comparator	synovial-fluid-derived exosomes	TMT quantitative proteomics	quantitative proteomics of SF-derived exosomes in inflammatory arthritis; potential inflammation-related exosomal markers	No glycan structures reported	OA used as comparator group for RA, axSpA and gout.	10.3389/fimmu.2022.800902
Chen et al., 2019	OA	synovial fluid	ELISA + Kellgren-Lawrence grading	MFG-E8	No glycan structures reported	Healthy controls; synovial fluid collected only from OA patients.	10.1177/0300060519862460

Chung et al., 2024	RA	saliva	ELISA + Luminex assay	alpha-1-antitrypsin	No glycan structures reported	Healthy controls.	10.3390/medicina60040605
Jasim et al., 2020	TMD myalgia	saliva	2-DE comparative salivary proteomics + LC-MS/MS	immunoglobulin J chain, immunoglobulin kappa light chain, alpha-amylase 1, alpha-amylase 2B, interleukin-1 receptor antagonist protein, cysteine-rich secretory protein 3, zinc-alpha-2-glycoprotein, chitinase-3-like protein 2	No glycan structures reported	Matched healthy pain-free controls.	10.3390/ijms21072569
Konar et al., 2025	OA/T2DM comorbidity	synovial fluid	LC-MS/MS comparative glycoproteomics + validation/ROC	thyroxine-binding globulin, alpha-1-antitrypsin, fibrinogen gamma chain, angiotensinogen	No glycan structures reported	OA patients without T2DM	10.1016/j.bbapap.2025.141067
Liao et al., 2018	OA vs RA vs traumatic arthritis	synovial fluid	SWATH-MS proteomics; ELISA validation	131 OA-associated differentially expressed proteins; 28 specifically regulated in OA. Key markers: complement C1r, DKK2; complement/coagulation proteins enriched.	No glycan structures reported	RA and traumatic arthritis comparators; no healthy SF controls.	10.3892/mmr.2017.8250

Lönsjö et al., 2025	OA	synovial fluid	proteomics / systems proteomic	OA-associated co-expression clusters included SERPINE1, CLEC11A, SPON1, FCGR3B, NCAM1, MMP2, MMP3, organization and immune-system pathways implicated	No glycan structures reported	Non-OA deceased donor controls.	10.3389/fimmu.2025.1523103
Mahendran et al., 2019	RA + PsA vs cadaveric controls	synovial fluid	label-free LC-MS/MS proteome and peptidome	fibrinogen chains, complement proteins, immunoglobulins, acute-phase/glycoprotein-related proteins	No glycan structures reported	Cadaveric synovial fluid controls.	10.1186/s12014-019-9243-3
Muller et al., 2023	OA	synovial fluid	mass-spectrometry proteomics in female OA vs non-OA	differentially regulated proteins in OA female synovial fluid; examples noted: presenilin and tenascin-C downregulated in OA	No glycan structures reported	Age-matched female non-OA controls.	10.3390/life13030605
PlsikovaMatejova et al., 2021	OA	synovial fluid	multiplex/biomarker assay	COMP, TIMP1, MMP3	No glycan structures reported	No healthy controls; early OA vs end-stage OA only.	10.1177/1947603520946385
Rydén et al., 2024	early OA; late OA	synovial fluid	DIA-MS proteomics; differential network analysis	human SF proteome/network analysis of early molecular OA; protein	No glycan structures reported	Healthy SF controls / mildly degenerated	10.1016/j.mcpro.2024.100785

				networks/pathways related to signal transduction, immune system, hemostasis and ECMorganization		knees / late-stage OA.	
Shao et al., 2023	TMJ-OA	synovial fluid	ELISA/clinical correlation	HMGB1	No glycan structures reported	TMJ internal derangemen t comparator; no healthy controls.	10.1186/s12891-023-06208-0
Takeshita et al., 2016	RA	synovial fluid	immunoprecipitation + antibody-overlay lectin microarray + Western blot/sialidase	MMP3	Core 1 O-glycans (T antigen); variable α2,6-sialylation of GalNAc; sialylation-dependent masking of T antigen	OA synovial fluid comparator for RA SF; validation RA serum cohorts.	10.1186/s13075-016-1013-2
Wu et al., 2024	OA	synovial fluid exosomes	iTRAQ quantitative proteomics; ELISA/ROC validation	C3, C4B, APOM, MMP3, DPYSL2	No glycan structures reported	Healthy controls / healthy SF-exosome controls.	10.1186/s13018-024-05336-0
Karalilova et al., 2018	OA	synovial fluid	ELISA + ultrasound (GS/PD)	YKL-40 (CHI3L1)	No glycan structures reported	Healthy age-matched serum controls; no healthy SF controls.	10.11152/mu-1247

Kazakova et al., 2017	RA	synovial fluid	ELISA	YKL-40 (CHI3L1)	No glycan structures reported	Age-matched healthy serum controls (n = 40); no healthy SF controls.	10.1515/folmed-2017-0013
Bei-Bei et al., 2026	RA	human joint/synovial fluid	cytokine signaling assay; soluble-factor experiments	IL6 trans-signaling / soluble IL6R / sgp130 axis; TGF-β pathway readouts	No glycan structures reported	Non-D2T RA, OA, and ankylosing spondylitis joint-fluid comparator groups.	10.1016/j.cyto.2026.157113
Bhattacharjee et al., 2016	RA	human synovial fluid	deep synovial-fluid proteome cataloguing by MS	956 proteins catalogued in RA synovial fluid; dataset supports biomarker discovery and comparison with plasma/synovial compartments.	Not a glycomics study; no individual glycan structures reported.	No control/comparator group; pooled RA synovial fluid samples only.	10.1186/s12014-016-9113-1
Birkelund et al., 2020	RA; spondyloarthritis comparator	human synovial fluid	LC-MS/MS proteomics	MMP3, S100A8/A9, complement and acute-phase proteins, immunoglobulin-related proteins	No glycan structures reported	Spondyloarthritis (SpA) patients served as the comparator group; no healthy SF	10.1186/s12014-020-09292-9

						controls.	
Flowers et al., 2020	OA	human synovial fluid	lubricin O-glycomics/glycoproteomics; functional galectin-3 interaction	Synovial lubricin/PRG4 investigated as mucin-type glycoprotein altered in OA.	Decrease of core 2 O-glycans on lubricin in OA; reduced galectin-3-mediated crosslinking.	Age- and sex-matched non-OA postmortem SF controls (n = 7) for O-glycomic analysis.	10.1074/jbc.RA120.012882
Harden et al., 2019	OA	human synovial fluid	multiplex inflammatory biomarker profiling	MMPs, cytokines/chemokines; macrophage/neutrophil-associated proteins	No glycan structures reported	No healthy/non-OA SF controls; analyses were performed within an OA cohort across inflammatory and severity measures.	10.1186/s13075-019-1923-x
Huang et al., 2020	OA	human synovial fluid	LC-MS/MS glycopeptide analysis; lectin/PAS/ELISA assays	PRG4/lubricin; cathepsin G-related PRG4 fragments	Mucin-type O-glycans on PRG4; PNA-reactive Galβ1-3GalNAc (T-antigen/core 1) structures;	No healthy/non-OA SF comparator; OA SF cohort only.	10.1038/s41598-020-61161-5

					glycopeptides detected	Lubricin incubated without cathepsin G served as an experimental assay control.	
Chen et al., 2023	OA	synovial fluid	IHC + flow cytometry/FACS/MACS + ELISA	DPP4/CD26	No glycan structures reported	OA progressors (n = 29) vs non-progressors (n = 28) during 3-year follow-up; no healthy SF controls.	10.1002/art.42455
Kobayashi et al., 2017	TMD	human synovial fluid	enzyme-linked immunosorbent assay (ELISA)	IL1β, IL6, IL8, TNF-α and other molecular inflammatory biomarkers	No glycan structures reported	No healthy control group; biomarker levels were correlated with clinical parameters within 28 TMD patients.	10.3892/mmr.2017.7012

Kumar et al., 2023	OA	synovial fluid	protein assay/ELISA correlated with KL severity	MMP1, MMP3, MMP9	No glycan structures reported	No healthy/non-OA controls; comparisons /correlations were performed across radiographic OA severity grades.	10.1007/s43465-023-00974-8
Mabey et al., 2016	OA	human synovial fluid	multiplex/ELISA biomarker assay correlated with radiographic OA	MMP3, COMP, HA-related and inflammatory/cartilage markers	NR; glycan structures not reported; HA is a glycosaminoglycan component, not a glycoprotein	Healthy controls (n = 14) for plasma comparisons ; no healthy SF controls.	10.3109/1354750X.2016.1171907
Nielsen et al., 2023	RA	synovial fluid	soluble marker assay + SF-FLS/osteoclast cultures	galectin-3	NR	Healthy controls (n = 48) for plasma comparisons ; chronic RA SF cohort had no healthy SF controls.	10.1080/03009742.2021.1992860
Ogawa et al., 2017	OA	synovial fluid	ELISA + radiographic/clinical assessment	lubricin (PRG4)	NR	No healthy SF controls; 61 OA knees spanning	10.1080/14397595.2016.1209829

						several disease stages were analyzed.	
Ornek Akdogan et al., 2023	TMD	saliva	ELISA + mandibular condyle fractal-dimension analysis	TNF-a. IL1b and MMP3	NR	Healthy controls (n = 34) compared with TMD patients (n = 34).	10.1016/j.jormas.2023.101417
Sarkar et al., 2020	RA	human synovial fluid cells	2D-DIGE/MALDI-TOF/TOF proteomics; validation	TXLNA / alpha-taxilin	NR; glycan structures not reported	OA patients served as SF/SF-cell controls for RA; healthy controls were included for plasma/blood analyses.	10.1155/2020/4897983
Weitoft et al., 2017	RA	human synovial fluid	Serum/SF biomarker assays + radiographic knee assessment	PTX3, TNF, IL6, VEGF, MMP3, COMP	NR; no glycan structures reported	Seropositive vs seronegative RA subgroups; no healthy SF control group.	10.1080/03009742.2016.1244288

Yaman et al., 2020	TMD	saliva + synovial fluid	biomarker concentration comparison/correlation assay	MMP2, chemerin	NR	No healthy controls; paired saliva and SF samples were obtained from 32 TMD patients.	10.1007/s00784-020-03310-8
