# Supplementary material for: Extracellular Matrix Remodeling and Glycoprotein Biomarkers Across Osteoarthritis, Rheumatoid Arthritis, and Temporomandibular Disorders: A Systematic Review and Integrative Analysis
Source: Int J Mol Sci. 2026 Sep 30;27(19):8764. doi: 10.3390/ijms27198764 (PMC13664865; doi:10.3390/ijms27198764)
Supplement: Supplementary file 1 [file ijms-27-08764-s001.zip › Table S2.pdf]

Table S2. Study-level justification for adapted ROBINS-I judgments.

Reference	Overall	Key rationale
Afshari et al., 2025	Moderate	Strong glycoproteomic/FILA methods and defined OA/control plasma comparison, but healthy SF unavailable, small MS discovery set, and residual confounding by BMI/clinical characteristics possible.
Ali et al., 2022	Moderate	Controls and OA stages were defined, but sample sizes were small, control SF came from deceased donors, early OA samples partly differed in collection/dilution, and exploratory co-expression analyses increase reporting bias risk.
Arvikar et al., 2021	Moderate	Appropriate clinical comparators and standardized multiplex assays; residual confounding by periodontal status, RA treatment/status and oral hygiene remains possible; SF available only for RA subset.
Äyräväinen et al., 2018	Moderate	Healthy controls and longitudinal RA sampling improve validity, but periodontal inflammation, RA treatment and disease duration are important confounders and follow-up attrition is possible.
Barreto et al., 2019	Moderate	Biologically coherent mechanistic work and direct LUM measurement; selection of surgical/available SF samples and limited clinical adjustment create moderate confounding/selection concerns.
Bhattacharjee et al., 2016	Serious	Deep LC-MS/MS plus lectin enrichment produced a useful RA SF catalogue, but 20 RA samples were pooled and no comparator group was analyzed, precluding individual-level variance/confounder assessment and limiting biomarker inference.
Birkelund et al., 2020	Serious	Clinically defined RA/SpA cohorts, standardized LC-MS/MS and FDR control are strengths; however, disease-group biological/demographic/treatment differences and absence of healthy SF leave important residual confounding.
Flowers et al., 2020	Moderate	Direct O-glycan profiling with age/sex-matched controls is strong, but the glycomic cohort was small, postmortem controls differed from clinical OA sampling, and MRM glycan estimates were semiquantitative.
Haraden et al., 2019	Serious	No non-OA comparator, a small OA cohort, lavage/direct aspiration differences, many biomarkers below detection and imputation; useful for inflammatory-endotype hypothesis generation but high bias for biomarker effect estimates.
Huang et al., 2020	Serious	Mechanistic experimental evidence for cathepsin-G-mediated lubricin degradation is strong, but the clinical OA SF component lacks a disease-free comparator and therefore provides limited disease-specific observational inference.
Huang et al., 2022	Moderate	Direct glycosylation/cytokine linkage and mechanistic stimulation data; control SF is cadaveric and some analyses are correlation-based with limited adjustment.
Huang Y et al., 2022	Serious	Disease groups were clinically classified and TMT proteomics used, but heterogeneous active diseases, OA comparator rather than healthy control, pooled samples for discovery and many pairwise omics contrasts raise bias risk.
Chen et al., 2019	Moderate	Large control group and ELISA measurement; SF inference lacks healthy SF controls and observational severity correlations are vulnerable to residual confounding.

Chen et al., 2023	Moderate	A longitudinal OA progression cohort and mechanistic validation strengthen the evidence, but observational confounding, loss from 65 baseline to 57 follow-up samples, and multiple exploratory analyses limit prognostic inference.
Chung et al., 2024	Moderate	Pilot case-control design with objective assays; small control group and age differences between RA and controls increase confounding risk; diagnostic threshold requires external validation.
Jasim et al., 2020	Moderate	Matched controls and DC/TMD classification support validity; small sample, 2-DE spot selection, one outlier exclusion and lack of independent validation keep risk moderate.
Karalilova et al., 2018	Serious	Useful YKL-40/ultrasound correlation, but serum controls only and no healthy SF controls; an active OA clinic cohort and limited adjustment leave substantial confounding for disease-specific biomarker inference.
Kazakova et al., 2017	Serious	ELISA measurements are appropriate, but synovial analyses were confined to active RA without healthy SF controls and limited adjustment for treatment/disease-activity confounders, producing serious risk for diagnostic or causal inference.
Kobayashi et al., 2017	Serious	Human TMD SF and mechanistic synovial-cell experiments support plausibility, but the clinical component was a small, predominantly female case-only cohort without a non-TMD comparator and used potentially variable push-pull SF sampling.
Konar et al., 2025	Serious	Relevant comparator groups and validation, but diabetes/OA severity, BMI, medication and trauma controls may confound glycoproteins; very small LC-MS discovery and candidate selection/ROC risk optimism.
Kumar et al., 2023	Serious	Defined OA criteria and transparent sampling are strengths, but age, BMI and symptom duration correlate with the same SF biomarkers while analyses are mainly unadjusted severity correlations; no non-OA SF comparator was included.
Liao et al., 2018	Serious	SWATH-MS and ELISA validation strengthen measurement; traumatic arthritis is not a true healthy control, groups were small and clinically heterogeneous, and late-stage surgical sampling limits generalizability.
Lönsjö et al., 2025	Moderate	Controls without knee OA and high-multiplex assay; deceased vs surgical samples and small sample size introduce selection/sampling concerns; exploratory network reporting remains moderate risk.
Mabey et al., 2016	Moderate	Age/gender-matched controls and standardized multiplex assays support plasma comparisons, but healthy controls did not provide SF and multiple cytokines were explored in a modest cohort without a prespecified analytical hierarchy.
Mahendran et al., 2019	Serious	Age-matched cadaveric controls and multiple-testing correction used; small cohorts, cadaveric-vs-clinical SF differences and combining IA subtypes increase confounding and selection bias risk.
Muller et al., 2023	Moderate	Sex-specific design and non-OA controls; small study, non-OA control indications and limited validation leave moderate confounding/selection and measurement concerns.
Nielsen et al., 2023	Moderate	Clinical galectin-3 measurements were reinforced by functional inhibition assays, but the chronic-RA SF cohort was small, healthy controls were plasma-only, and treatment/disease-stage confounding remains possible.

Ogawa et al., 2017	Serious	Standardized ELISA and clinical measures were used, but the study was a selected surgical OA cohort without a healthy/non-OA SF comparator and relied on cross-sectional correlations.
Ornek Akdogan et al., 2023	Serious	Balanced TMD/healthy groups and standardized saliva collection are strengths, but psychological/oral confounding was not fully adjusted and several biomarkers plus imaging/psychological outcomes were explored in a modest cohort.
Plsikova Matejova et al., 2021	Serious	Useful stage comparison with multiplex assay, but no healthy/non-OA comparator and stage differences may be confounded by age, sex, medication and surgery selection.
Rydén et al., 2024	Serious	Balanced age/sex groups and defined donor grading; however high-dimensional network analysis with small n, feature filtering by fold change and exploratory modeling create serious reported-result bias risk.
Sarkar et al., 2020	Serious	RA/OA comparator groups and multi-platform validation strengthen α-taxilin evidence, but discovery used small SF-cell proteomic samples and the candidate was selected post hoc from a high-dimensional screen, creating serious selective-reporting/optimism risk.
Shao et al., 2023	Serious	Standard ELISA and clinical correlations, but comparator is another TMJ disease rather than healthy SF, retrospective sampling and disease severity differences may confound biomarker associations.
Takeshita et al., 2016	Moderate	Specific glycoform assay, validation cohort and OA SF comparator; residual confounding by treatment and disease activity, plus biomarker-index derivation, keep overall risk moderate.
Weitoft et al., 2017	Moderate	A relatively large RA cohort and standardized assays support seropositive/seronegative comparisons, but disease activity, treatment and severity may confound subgroup differences and no healthy SF comparator was available.
Wu et al., 2024	Serious	Discovery plus external/public dataset comparison and validation; high-dimensional iTRAQ with marker selection and ROC validation likely optimistic, and exosome isolation/healthy-control comparability require caution.
Yaman et al., 2020	Serious	Paired saliva/SF sampling is useful for matrix correlation, but the clinic-based study had no disease-free comparator; disease-biomarker claims therefore rest on within-patient matrix differences and exploratory correlations.
Yang et al., 2026	Serious	Defined D2T/non-D2T RA groups and disease-fluid comparators support the mechanism, but very small cohorts, treatment-history/disease-activity differences and multiple exploratory functional endpoints leave substantial residual confounding.
